# Supporting self-management of low back pain with an internet intervention in primary care: a protocol for a randomised controlled trial of clinical and cost-effectiveness (SupportBack 2)

**DOI:** 10.1136/bmjopen-2020-040543

**Published:** 2020-08-20

**Authors:** Adam W A Geraghty, Lisa Roberts, Jonathan Hill, Nadine E Foster, Lucy Yardley, Elaine Hay, Beth Stuart, David Turner, Gareth Griffiths, Frances Webley, Lorraine Durcan, Alannah Morgan, Stephanie Hughes, Sarah Bathers, Stephanie Butler-Walley, Simon Wathall, Gemma Mansell, Linda Leigh, Paul Little

**Affiliations:** 1 Primary Care, Population Sciences and Medical Education, University of Southampton, Southampton, Hampshire, UK; 2 School of Health Sciences, University of Southampton & University Hospital Southampton NHS Foundation Trust, Southampton, Hampshire, UK; 3 Primary Care Centre Versus Arthritis, School of Primary, Community and Social Care, Keele University, Keele, Staffordshire, UK; 4 Department of Psychology, University of Southampton, Southampton, Hampshire, UK; 5 School of Psychological Science, University of Bristol, Bristol, Bristol, UK; 6 Keele Clinical Trials Unit, School of Primary, Community and Social Care, Keele University, Keele, Newcastle, UK; 7 School of Life and Health Sciences, Aston University, Birmingham, UK; 8 Keele Clinical Trials Unit, Keele University, Keele, Staffordshire, UK; 9 Patient and Public Involvement Representative, University of Southampton, Southampton, UK

**Keywords:** back pain, primary care, rehabilitation medicine

## Abstract

**Introduction:**

Self-management and remaining physically active are first-line recommendations for the care of patients with low back pain (LBP). With a lifetime prevalence of up to 85%, novel approaches to support behavioural self-management are needed. Internet interventions may provide accessible support for self-management of LBP in primary care. The aim of this randomised controlled trial is to determine the clinical and cost-effectiveness of the ‘SupportBack’ internet intervention, with or without physiotherapist telephone support in reducing LBP-related disability in primary care patients.

**Methods and analysis:**

A three-parallel arm, multicentre randomised controlled trial will compare three arms: (1) usual primary care for LBP; (2) usual primary care for LBP and an internet intervention; (3) usual primary care for LBP and an internet intervention with additional physiotherapist telephone support. Patients with current LBP and no indicators of serious spinal pathology are identified and invited via general practice list searches and mailouts or opportunistic recruitment following LBP consultations. Participants undergo a secondary screen for possible serious spinal pathology and are then asked to complete baseline measures online after which they are randomised to an intervention arm. Follow-ups occur at 6 weeks, 3, 6 and 12 months. The primary outcome is physical function (using the Roland and Morris Disability Questionnaire) over 12 months (repeated measures design). Secondary outcomes include pain intensity, troublesome days in pain over the last month, pain self-efficacy, catastrophising, kinesophobia, health-related quality of life and cost-related measures for a full health economic analysis. A full mixed-methods process evaluation will be conducted.

**Ethics and dissemination:**

This trial has been approved by a National Health Service Research Ethics Committee (REC Ref: 18/SC/0388). Results will be disseminated through peer-reviewed journals, conferences, communication with practices and patient groups. Patient representatives will support the implementation of our full dissemination strategy.

**Trial registration number:**

ISRCTN14736486.

**Strengths and limitations of this study**

- The SupportBack 2 trial is a large multicentre randomised trial that will determine the additional benefit, over usual primary care, of an internet-based approach that supports self-management of patients with low back pain (LBP) in UK primary care.
- The trial is designed to investigate the effectiveness of an internet intervention in addition to usual primary care, both with and without telephone physiotherapist support.
- A full mixed-methods process evaluation will be carried out to inform a logic model and ‘theory of change’ for the interventions.
- Inclusion is limited to those with LBP who have access to the internet and are able to communicate in English without assistance.

## Introduction

Low back pain (LBP) has a lifetime prevalence of up to 85%^1^ and is the greatest single cause of years lived with disability globally.^2^ LBP is primarily managed in primary care,^3^ where first-line recommendations are to self-manage and remain physically active.^4^ Supporting effective behavioural self-management of LBP is increasingly important; the most recent guidelines place less emphasis on pharmacological and surgical treatments.^5^ General practitioners (GPs) are unlikely to have the training or the capacity to support behavioural self-management, and access to specialist musculoskeletal (MSK) services can be variable.^6^ New roles such as First Contact Physiotherapists in general practice are emerging, but implementation is at an early stage.^7^ Internet interventions may offer a route to rapidly scalable behavioural support for patients with LBP, however, their effectiveness in UK primary care needs to be determined.

Internet interventions are typically automated, interactive, tailored interventions that make use of multimedia formats to deliver behavioural change strategies online.^8^ Internet interventions are one form of a broader category of digital interventions that draw on digital technologies including the internet, mobile devices and activity sensors.^9^ A recent systematic review of digital interventions for LBP highlighted substantial heterogeneity in intervention delivery, duration and outcomes, making it difficult to draw conclusions regarding effectiveness.^10^ Since the publication of this review, there has been a focus on mobile apps for LBP: a German study has shown that a mobile app delivering multidisciplinary self-management support for patients with LBP recruited via online advertising significantly reduced pain at 12-week follow-up, compared with a 6-week course of exercise delivered by physiotherapists plus online education.^11^ An ongoing European programme of work seeks to determine the effectiveness of a mobile app-based digital decision support self-management programme (selfBACK) for patients recently consulting in primary care for LBP.^12^ While mobile apps show potential, internet interventions likely have an accessibility advantage; they can be accessed from any device with an internet connection (eg, desktop, laptop, tablet, mobile phone).

SupportBack is an internet intervention designed to support patients to self-manage their LBP following consultation in primary care.^13 14^ It was developed using evidence and theory in combination with the person-based approach, where systematic qualitative research is integrated throughout development.^15 16^ SupportBack is designed to be as accessible as possible, both in presentation style and in target; it can be used by people with both acute and persistent LBP. SupportBack has been developed to be used in addition to usual care, either as a stand-alone internet intervention or in combination with physiotherapist telephone support. A randomised controlled feasibility trial demonstrated both the feasibility of trial procedures and the effective delivery of the intervention and telephone support.^14^ In a nested qualitative study within the feasibility trial, Geraghty *et al*^15^ found that patients were broadly positive about the intervention; they suggested that it provided reassurance while supporting becoming more physically active as a primary pain management strategy.

The aim of the present full randomised controlled trial (RCT) is to determine the clinical and cost-effectiveness of the SupportBack internet intervention, delivered in addition to usual care with and without physiotherapist telephone support, in reducing LBP-related physical disability in UK primary care.

## Methods

### Design

A three-parallel arm (1:1:1), multicentre RCT is being conducted to determine the clinical and cost-effectiveness of an internet intervention for patients with LBP in primary care. Participants will be followed up at 6 weeks, 3, 6 and 12 months.

### Study setting

The trial is being carried out with patients from 140 to 180 general practices across the UK. Patients access the intervention through their own devices with internet access (eg, a desktop, laptop, tablet, mobile phone) at a location that is convenient for them (eg, at home or at work). If allocated to receive telephone physiotherapist support, this support is delivered wherever is convenient for the patient. A list of patient identification centres is available from the trial team on request.

### Eligibility criteria

#### Inclusion criteria

- Aged 18 and above.
- Current LBP (have experienced pain in the last week) with or without sciatica.
- Access to the internet and an active email address.
- Ability to read/understand English without assistance.
- Ability to provide informed consent.

#### Exclusion criteria

- Signs and symptoms in a patient with LBP that indicate potential serious spinal pathology such as infection, malignancy, fracture, inflammatory back pain, progressive neurology and/or cauda equina.
- Have had spinal surgery in the past 6 months.
- Pregnancy.
- Taken part in the prior SupportBack feasibility study.

### Identification, recruitment and screening

Two recruiting centres, Southampton and Keele (each with a team of telephone support physiotherapists) are working with National Institute for Health Research Clinical Research Networks to facilitate the recruitment of general practices. Potentially eligible participants will be identified in one of two ways:

1. Patients who have consulted with LBP in the last 2 months will be identified by general practice staff from computerised records of consultations. Practices will be asked to repeat the searches approximately three times, or until the target number of patients per practice has been reached. Resulting lists of patients identified by the search will be screened by a practice GP who will rule out patients based on aspects of the eligibility criteria that can be determined from patient notes.
2. During a patient consultation and on entering a relevant diagnostic or symptom Read code into the patient electronic medical record, GPs will be prompted about the trial and patient eligibility by an automated ‘pop-up’ screen activated by the Read code. GPs will then screen for eligibility (using the inclusion/exclusion criteria listed) and patients identified as suitable will have their medical record electronically tagged. A download of ‘tagged’ patients will occur regularly, anticipated to be every 2 weeks. This method will be used in practices where possible. Participating general practices not implementing the ‘pop up’ Read code method can identify potential patients during consultation. Having considered eligibility the GP or nurse practitioner will provide the patient with an invitation pack.

Patients identified either by a medical records review or general practice consultation are mailed a study pack including an invitation letter from the GP, participant information sheet, reply slip, screening questions and prepaid envelope. Interested patients return the reply slip and screening questions using the prepaid envelope to the research team. Screening consists of two questions regarding current LBP and access to the internet, followed by three safety questions listing symptoms that may indicate serious spinal pathology. Patients who answer ‘yes’ to the first two questions, and ‘no’ to all safety questions, are considered eligible. For those who complete the screening questions and fail safety screening, a physiotherapist contacts the patient to make an appropriate clinical recommendation on hearing a further description of the symptoms. Those who fail the screening are documented on a screening log maintained by the research team. All patients considered eligible for the trial are assigned a unique participant identification number and sent a link to the study website, to complete consent, baseline questionnaires and be randomised. Recruitment opened in November 2018 and is expected to close in December 2020, with data collection completing approximately 12 months later in December 2021.

### Randomisation, allocation and blinding

**Figure 1:**
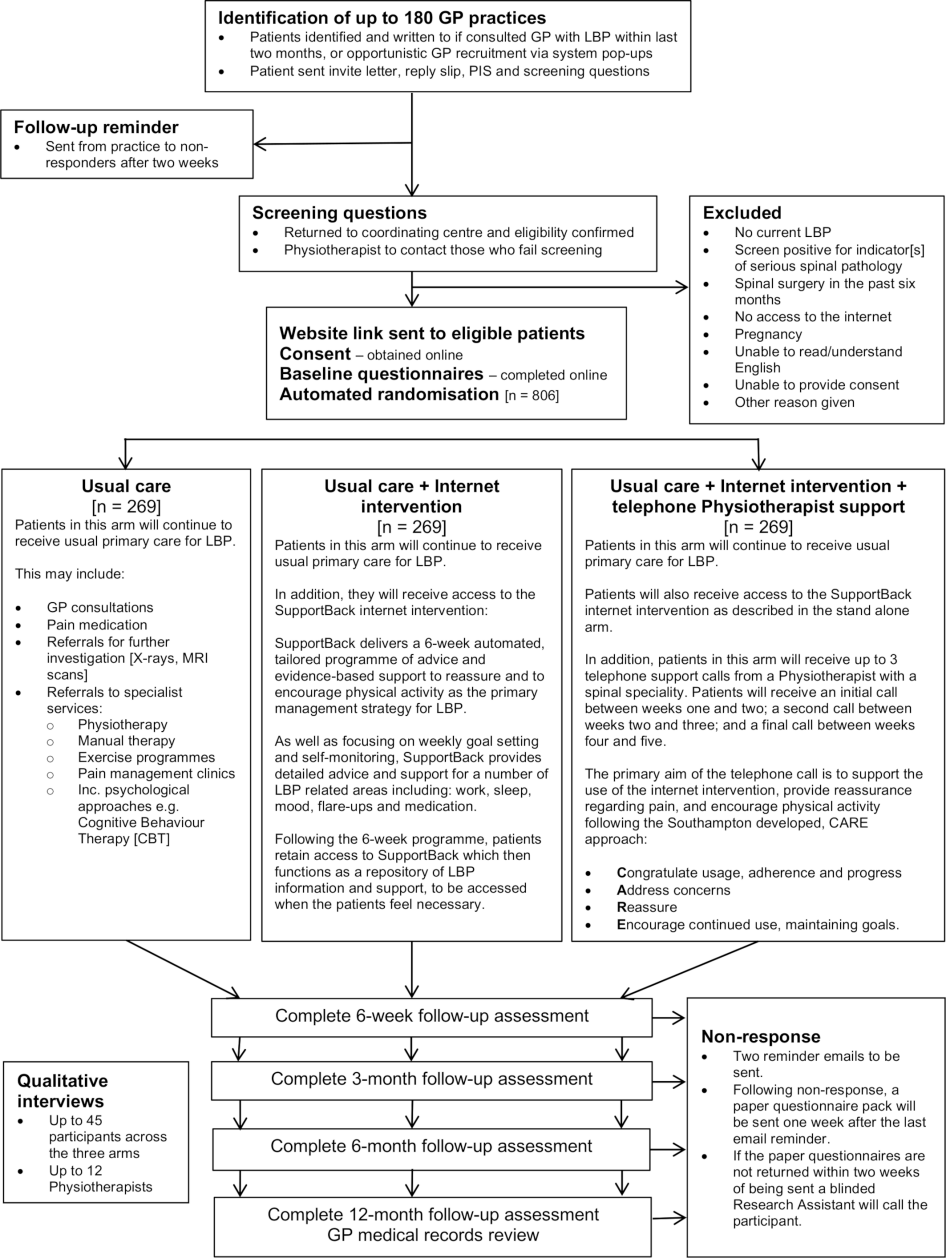
Flow through the trial. CARE, Congratulate, Ask, Reassure, Encourage; GP, general practitioner; LBP, low back pain; PIS, Patient Information Sheet.

The randomisation process for this trial is fully automated. The intervention and data collection software automatically generates the randomisation sequence, and a computer-generated algorithm block randomises participants to the trial groups. Participants are being stratified by trial recruiting centre and level of physical function: a score of less than four on the Roland Morris Disability Questionnaire (RMDQ^17^) is being used to denote a lower level of self-rated physical disability. As the automated software randomises patients, the randomisation sequence is concealed from the trial team. Patients are automatically informed of their group allocation through the internet intervention software. As patients are engaging with a behavioural intervention, they are not blind to allocation. The majority of data will be collected online, or by post. Telephone calls are used to collect primary outcome data where there has been no response to online and postal follow-up. The callers are blind to group allocation. The statisticians conducting the analyses will remain blind to group allocation. The health economist will conduct the majority of analysis blinded to group, however, estimates of total cost require the addition of costs specific to the provision of the interventions so will become unblinded at this point. Figure 1 details patient flow through the trial.

### Interventions

#### Usual care

Participants randomised to this arm will continue to receive unrestricted usual primary care for LBP. Current National Institute for Health and Care Excellence recommendations for primary care management of LBP suggest assessment to rule out specific spinal pathology and use of risk stratification tools (eg, STarT Back^18^). Guidelines with regard to pharmacotherapy recommend Non-steroidal anti-inflammatory drugs (NSAIDs) at the lowest effective doses for the shortest period of time. Paracetamol and opiates are not recommended for routine use. Care may also include referrals for physiotherapy and psychological interventions such as cognitive–behavioural therapy. Despite guidelines, there is likely to be a variety in what is provided for patients as part of usual care over the trial period from no further contacts, to referrals to physiotherapy and pain clinics. Consequently, healthcare resource use will be documented and form a central part of our health economic analysis.

#### Usual care+internet intervention

Participants randomised to this arm will continue to receive unrestricted usual primary care. In addition, they will have access SupportBack. SupportBack is a multisession, interactive internet intervention that provides participants with accessible information, advice and tools to support the effective self-management of LBP. The SupportBack intervention (including telephone support) has been extensively described elsewhere.^13 15^ In brief, the central components of the intervention include graded goal setting, self-monitoring and tailored feedback to encourage physical activity (PA)/exercise increases or maintenance. SupportBack also provides educational modules regarding pain and LBP-related topics (relieving pain; flare ups; work; sleep; mood; daily living). Throughout the digital material, there is a focus on supporting motivation for behavioural change: Techniques employed include reassuring about consequences of movement, modelling of use of activity as a primary pain management strategy and using automation to deliver positive feedback through reassurance regarding consequences. These techniques were combined with a person-based approach in the development of SupportBack, where the application of such approaches was guided by systematic in-depth qualitative research with patients with LBP.^16^

Practically, patients can access SupportBack from any device with an internet connection from wherever is most convenient for them. SupportBack consists of six sessions, and patients are encouraged to log in and use one session per week. Automated reminders adhere to this schedule. The first session highlights the centrality of PA in managing LBP, and supports patients to set goals to either walk more, or engage with a range of gentle back exercises of their choice. Goal options are tailored and are based the extent that patients report their LBP obstructs their day-to-day activities. The further sessions feature self-monitoring and feedback regarding their progress with walking or exercise goals, combined with encouragement from SupportBack to continue. After the first session, patients can unlock one further module per week on topics such as sleep, mood and work. These build into a personal repository, that alongside weekly goals, can be accessed at any time. If engaged with weekly, the tailored, interactive part of the intervention will last 6 weeks. Following completion of all the sessions, SupportBack converts into static resource where all activities/exercises and modules can be accessed for the duration of the trial.

#### Usual care+internet intervention+telephone physiotherapist support

Participants randomised to this arm will also continue to receive unrestricted usual primary care, with access to the SupportBack internet intervention. In addition, these participants will also receive up to 1 hour of physiotherapist support over the telephone (the first call can be up to 30 min, with two follow-up calls of up to 15 min, over the 6-week intervention period). At both centres (Southampton and Keele) support is provided by MSK physiotherapists working in the National Health Service (NHS).

The objectives of the telephone contact are to encourage the use of the SupportBack intervention, provide reassurance regarding LBP and encourage adherence to PA goals. The physiotherapists are asked to closely adhere to a standardised content checklist for each phone call. The checklist follows the Congratulate, Ask, Reassure, Encourage approach,^19^ developed specifically to guide support for digital interventions. Drawing on existing clinical skills, it ensures a general supportive approach and requires minimal training (all support physiotherapists attended a 2-hour training session ahead of the trial). While telephone physiotherapists are able to address individual participant concerns, they are asked to avoid additional individualised participant assessment and treatment recommendations beyond the internet intervention content. Physiotherapists complete a checklist for each call. Checklists are returned to the trial coordinating centre, where intervention fidelity will be assessed.

### Measures

All measures and time points for collection are listed in table 1.

**Table 1:** Outcomes and measures used in the trial

Domain	Measure	Time point
Function (primary outcome)		
LBP-related physical function	Roland Morris Disability Questionnaire.^17^	Baseline, 6 weeks, 3, 6, 12 months. All arms.
Pain		
Pain intensity	Pain index (Numerical Rating Scales measuring current, average and least pain over the last 2 weeks).^22^	Baseline, 6 weeks, 3, 6, 12 months. All arms.
Pain duration	Time since last pain-free month.^45^	Baseline. All arms.
Troublesomeness of pain	Troublesome days in pain over the last month(developed from days in pain measure.^23^	Baseline, 6 weeks, 3, 6, 12, months. All arms.
Risk of persistent pain-related disability	STarT Back tool.^24^	Baseline, 12 months. All arms.
Psychological processes related to pain		
Fear of movement	Tampa Scale for Kinesiophobia.^25^	Baseline, 12 months. All arms.
Catastrophising/negative orientation towards pain	Pain Catastrophizing Scale.^26^	Baseline, 12 months follow-up All arms.
Confidence in ability to manage pain	Pain Self-Efficacy Questionnaire.^27^	Baseline, 6 weeks, 12 months. All arms.
Self-efficacy for managing LBP	Single item from Musculoskeletal Health Questionnaire.^46^	Baseline, 6 weeks, 3, 6, 12 months. All arms.
Outcome expectation	Expectancy question from Credibility and Expectancy Questionnaire modified for LBP modification.^28 47^	Baseline, all arms. following session one of SupportBack Internet intervention arms only.
Mental health	Patient Health Questionnaire-4^29^ depression and anxiety measure.	Baseline, 12 months. All arms.
Physical activity/adherence		
General physical activity	Godin leisure-time exercise questionnaire.^30^	Baseline, 12 months. All arms
SupportBack-related physical activity	Single item measure developed for the trial.	Baseline, 6 weeks, 3, 6, 12 months. All arms.
Adherence to back-specific activity	Item developed for this trial, based on previous behavourial adherence measures.^32^	12 months. All arms
Difficulties with intervention recommendations	Problematic Experiences of Therapy Scale.^33^	12 months. Internet intervention arms only.
Satisfaction and enablement		
Satisfaction with care received for LBP	Single satisfaction item developed for trial.	6 weeks. All arms.
Enablement	Patient Enablement Instrument.^48^	6 weeks, 12 months. All arms.
Health related quality of life, healthcare resource use and occupational status		
Health-related quality of life	ED-5D-5L.^34^	Baseline, 6 weeks, 3, 6, 12 months. All arms.
Use of over-the-counter (OTC) medication for LBP	Single item measuring self-reported OTC medication usage for LBP.	Baseline, 6 months, 12 months. All arms.
Participant borne costs	Participant reported health resource use questionnaire developed for this study.	Baseline, 6 months, 12 months. All arms.
NHS healthcare resource use (specific to back pain, and general)	General practice medical notes review and participant reported healthcare resource use questionnaire developed for this trial.	Baseline, 6 months, 12 months. All arms.
Occupational impact of LBP	Brief occupational questionnaire developed for this trial.	12 months. All arms.
Use of internet resources		
Use of internet resources	Single item regarding use of internet resources for LBP over trial period.	12 months. All arms.

LBP, low back pain; NHS, National Health Service.

#### Primary outcome

The primary outcome in this trial is LBP-related physical function measured with the RMDQ.^17^ Function forms a central domain in the recommended core outcomes set for LBP trials.^20^ The RMDQ is a recommended measure of physical function^21^ and is commonly used in primary care LBP trials.

#### Secondary measures

Demographic data are being collected at baseline including age, sex, educational attainment, marital and occupational status. A range of secondary measures are being collected including pain intensity,^22^ number of troublesome days in pain^23^ and risk of pain related disability,^24^ Pain-related psychological variables are being measured including kinesiophobia (fear of movement),^25^ catastrophising,^26^ pain self-efficacy (SE),^27^ outcome expectations^28^ and symptoms of depression and anxiety.^29^ General PA is being measured with the Godin Leisure-time Exercise Questionnaire^30^ in this trial; the short-form International Physical Activity Questionnaire^31^ was used in the feasibility trial but produced unreliable data. We are also measuring intervention specific PA with a single item developed for this trial. Adherence to walking and PA goals are being measured with specifically developed items, based closely on measures previously used in a related behavioural trial^32^ and difficulties with the intervention are being measured using the Problematic Experiences of Therapy Scale.^33^

To support the health economic analysis health-related quality of life is being measured with the 5-level EQ-5D (EQ-5D-5L)^34^ All resources required to provide the internet intervention and the telephone support will be recorded. Details of NHS resource use will be recorded from general practice notes review. This will include both primary and secondary care contacts and will cover both general healthcare usage in addition to LBP specific care in the follow-up period. Additionally, LBP-specific medication use will be captured. There may also be differences in LBP related services paid for by study participants: for example, complementary or complementary medicine. Participants may also require time off work. Additionally, there may be under-reporting of LBP-specific resource use from medical records. These resources will be captured by means of a simple questionnaire administered at six and 12 months. The time-off work question and items relating to use of private healthcare will additionally be asked at baseline. All resources identified will be costed using appropriate local and national data, for example, NHS reference costs and Unit Costs of Health and Social Care. Occupational status is being measured with a brief questionnaire developed for this trial.

The internet intervention software automatically collects data on number of logins, page and module views and time spent in each login. This data will be used to explore adherence and user engagement to the digital component of the intervention.

### Sample size

The reported minimally clinical important difference (MCID) between groups for the RMDQ varies. A between group MCID of 2 or 3 points is commonly reported.^23 35 36^ However, it has been suggested that a difference of 1.5 may still be important, particularly in the context of low intensity interventions.^23^ Whether delivered as a stand-alone intervention or coupled with brief telephone support, SupportBack is a low intensity intervention with the potential to be rapidly scalable. Consequently, we considered a between group change of at least 1.5 to be a meaningful difference in this context. For our repeated measures primary outcome, a difference of 1.5 points on the RMDQ over the follow-up period of 12 months, assuming an SD of 5 in line with the feasibility study,^23^ gives an effect size of 0.30. Alpha will be set to 0.025 to allow both interventions to be independently compared with the usual care alone arm. Using four repeated measures (6 weeks, 3, 6 and 12 months), and assuming a correlation between repeated measures of 0.7% and 90% power, requires 215 participants per arm. Allowing for 20% lost to follow-up, this gives a total sample size of 806.

### Data collection and management

Data are primarily being collected online. The LifeGuide intervention and data system collects consent, baseline data including demographics and follow-up data across the four time points (6 weeks, 3, 6 and 12 months). When first sent a link to the system following screening, if patients do not log on within a week, they are emailed to check that they received the link and advised to look in their spam mail. If there is no response, one telephone call is attempted by the research team.

With regard to follow up protocol, where there is no response to the online follow-up questionnaire emails, two reminder emails and text messages will be sent. Following continued non-response, a paper questionnaire pack with a prepaid envelope will be sent 1 week after the last email/text reminder. If the paper questionnaires are not returned within 2 weeks of being sent, a blinded research assistant will call the participant to complete the primary outcome measure (RMDQ), quality of life questionnaire (EQ-5D-5L) and pain severity. If the participant is happy to continue, further measures from the questionnaire battery at the respective follow-up point will be collected in this manner. The full follow-up protocol with the telephone calls will be implemented at 6 weeks and 12 months follow-up points. These two follow-up points are considered most important, capturing initial and long-term response. Calling at all time points may lead to increased dropout at later time points. Follow-up at 3 and 6 months will include all the above steps except for the phone calls. All participants will receive a £5 voucher when asked to complete questionnaires at the more distant time points of 6 and 12 months. Examples of data collection forms can be provided by the trial team on request.

### Statistical methods

Quantitative analysis will begin following cleaning and inspection of the data. Descriptive analysis will be conducted to determine outliers and distributions of the data. Where necessary, if data are not normally distributed, transformations will be applied or another appropriate distribution used. The primary analysis for the RMDQ score will be performed using a multilevel mixed model framework with observations at 6 weeks, 3, 6 and 12 months (level 1) nested within participants (level 2). Results will be reported adjusting for baseline severity in function, stratification factors and any prespecified confounders. The model will use all the observed data and makes the assumption that missing RMDQ scores are missing at random given the observed data.

As there may not be a constant treatment effect over time, a treatment/time interaction will be modelled and included if significant (at the 5% level), with time treated as a random effect. An unstructured covariance matrix will be used.

Analysis of secondary outcomes will also be conducted using linear regression for continuous outcomes and logistic regression for dichotomous outcomes, again controlling for baseline symptom severity, stratification factors and any potential confounders. The structure and pattern of missing data will be examined, if appropriate, and a sensitivity analysis based on data imputed using a multiple imputation model presented. Data will be analysed on an intention-to-treat basis (they will be analysed as randomised). We will also undertake a complier-average causal effect analysis,^37^ which compares compliant participants in the intervention group, with those in the control group whose characteristics are similar enough to the intervention group compliers to suggest they too would have complied with the intervention, given the opportunity to do so. Compliance for these analyses in the intervention arm will be defined as completing at least session 1 of the internet intervention. Session 1 contains the central rationale for the intervention; that PA is primary in the management of LBP and provides instructions and advice on goal setting. The latter sessions follow a similar format to the first introductory module. With regard to the physiotherapist telephone support arm, we consider per protocol to be receiving at least two of the three planned phone calls. The telephone element is designed to be pragmatic with the necessary flexibility to fit patients’ requirements. However, receiving at least two of three calls indicates that support was delivered over time; an important aspect in the design and integration with the internet intervention.

It is not anticipated that there will be significant practice level (cluster) effects but this assumption will be tested by comparing a fixed effect model to a random effects model. If there are significant practice level effects then, the model will include a random effect for practice (random intercept) and participant (random intercept and slope on time) to allow for between participant and practice differences at baseline and between participant differences in the rate of change over time (if significant at the 5% level), and fixed effects for baseline covariates.

No interim analyses are planned. Full details of the analyses to be undertaken will be set out in the statistical analysis plan and approved by the trial steering committee (TSC). Our full statistical analysis plan will be published on the trial website in due course (https://www.southampton.ac.uk/medicine/academic_units/projects/supportback2.page).

#### Cost-effectiveness analysis

A ‘within-trial’ economic analysis will be conducted alongside the RCT to estimate the incremental cost-effectiveness of the SupportBack 2 interventions compared with usual care. The base case perspective will be that of the NHS, but other resources relevant to LBP will be collected to enable additional analysis from a societal perspective.

The main outcome measure in the economic evaluation will be the quality-adjusted life year (QALY), obtained from the EQ-5D-5L instrument using the published UK value set. In addition, a cost-effectiveness analysis will be carried out using the study primary outcome measure, that is, the cost per point change in back-related physical function measured using the RMDQ will be estimated. Both costs and effects will be estimated using multiple regression, to allow for potential confounders, such as baseline scores for EQ-5D-5L and RMDQ. Standard practice will be followed to calculate incremental cost-effectiveness ratios (ICERs), and present ICER(s) where any one option has both higher costs and increased effects compared with another. ICERs will show incremental cost per QALY or incremental cost per point improvement in RMDQ. Bootstrapping will be used to calculate cost-effectiveness acceptability curves. These will illustrate the effect of uncertainty on study results. Major assumptions made in the analysis will be tested by means of sensitivity analysis. In particular, assumptions made during the costing of the intervention such as the number of individuals who will be using the website will be explored. Similar methods to the main clinical analysis will be used to handle missing data, that is, analysis of patterns of missing data with multiple imputation methods employed if deemed appropriate. The proposed health economics analysis will be detailed in a health economics analysis plan (HEAP) which will be completed before analysis commences. The HEAP will be circulated for comment prior to the health economics analysis. Any digressions from the HEAP will be documented and justified in the final health economics report.

### Process evaluation

A process evaluation will be carried out following Medical Research Council guidelines on process evaluations of complex interventions.^38^ In order to provide a detailed understanding of the SupportBack intervention three aspects will be examined: Implementation, mechanisms of impact (mediators) and context (moderators). A mixed-methods approach will be used to explore these elements.

#### Implementation

Quantitative data describing trial implementation will be presented including number of practices recruited, patient eligibility (including reasons for declined participation where possible, and analysis of screen failures) and recruitment rates. The number of withdrawals from the trial per arm will be presented, along with numbers/percentages of drop-outs from the intervention who do not respond to follow up. Use of the internet intervention will be described by presenting automated data collected on number of logins and modules accessed for both the internet intervention and the intervention plus telephone physiotherapist support arm. With regard to the internet intervention plus telephone physiotherapist support arm, the number of support calls successfully made (and attempts contact the patient), along with the mean number per participant in this arm will be described.

Qualitative interviews will be conducted with up to 45 trial participants following the 3, 6 and 12 months follow-up points. Different participants will be interviewed at each time point, enabling us to explore how time since accessing the tailored weekly component of the intervention effects how suggestions are used and implemented in daily life. Interviews will also be conducted with the trial physiotherapists. Participants will be purposively sampled to ensure diversity in terms of age, sex and symptom severity (physical function, pain intensity and duration). Participants will also be sampled based on high and low usage of the internet intervention and high and low engagement with the telephone physiotherapist support. For participants, questions will focus on their experience of using the intervention, including telephone physiotherapist support and usual care. Interviews with the trial support physiotherapists will be designed to explore their experience of delivering the intervention, with a particular focus on barriers and facilitators, and determinants of successful exchanges.

#### Mechanisms of impact

A logic model of proposed mechanisms affecting LBP-related physical disability and pain outcomes for the SupportBack intervention has been developed (see ref.^14^). This model will be used as the basis of both quantitative and qualitative exploration of mechanisms. Quantitative analyses will focus on psychological and behavioural mechanisms influencing outcome following use of the interventions including expectancy, self-efficacy, PA, self-reported goal setting across the intervention and objective measures of intervention use (sessions completed, use of additional modules, for example, mood, sleep etc). In order to explore whether two core mechanisms’ (mediating variables) contribution to outcome is unique to the internet intervention arms, brief single items capturing SE and PA are being measured in all three arms (including usual care). SE and PA are being measured at baseline and in the outcome questionnaire sets at 6 weeks, 3, 6 and 12 months. Mediation analysis will be used to explore relationships between mediating variables and LBP-related physical function and pain intensity across the 12-month follow-up period. We will also explore the potential of multilevel modelling to examine mediating variables association with the outcome over time.^39^ Appropriate checks of the assumptions of causal modelling, such as exchangeability (no confounding), consistency, effect modification and temporality will also be carried out.^40 41^

Questions will be included in the qualitative interviews focusing on participants’ perceptions of how use of the SupportBack intervention and/or telephone support affected their LBP. This will enable the inductive exploration of participants’ views and triangulation of qualitatively derived theory on mechanism with our quantitative analysis. Similar questions will also be explored in the usual care arm, focusing on how elements of their usual care may have led to improvements in their LBP.

#### Context

The relationship between elements of participants’ context (moderators) and the effect of the interventions across the 12 months follow-up period will be explored. This will include variables such as LBP severity and duration at baseline, age, educational level and occupation status. Following the analysis of mechanisms, correlations and multiple regression (linear and logistic) will be used to explore relationships between moderating variables and LBP-related physical function and pain intensity. Qualitatively, the above aspects of participants’ context, including their own descriptions of their LBP history, will feed into analysis when exploring themes regarding participants use of the intervention and their perceptions of benefit.^42^

#### Qualitative analysis

Interview data collected regarding implementation, mechanisms and context will be transcribed verbatim, coded and analysed using an inductive thematic analytic approach.^43 44^ This will ensure participants’ qualitative data are not constrained by the direction of a particular theoretical model, and enable novel insights from qualitative work to be added into the theory-driven logic model. A key aspect of the qualitative analysis will entail exploring differences in accounts at different time points. This will enable us explore how time since the tailored weekly sessions impacts on the process of self-management.

### Data monitoring and confidentiality

The SupportBack 2 trial has a data monitoring and ethics committee (DMEC) composed of a statistician (chair) and two academic clinicians (Professor in Primary Care Research and Professor of Physiotherapy respectively). The DMEC reports to the TSC and is fully independent from the trial Sponsor with no competing interests. Interim descriptive analyses are prepared for the DMEC. The DMEC charter can be obtained from the research team on request.

All serious adverse events (SAEs) are reported to the lead clinical trial unit. The assessment of seriousness will be made by the participants GP or delegate. Assessment of causality will be made by the GP or delegate, and related or unrelated status will be determined. As the SupportBack intervention provides reassurance and encourages gentle activity within the participants’ own limits, there are no ‘expected’ SAEs documented.

All patient data are being kept in strict confidence and managed in accordance with the Data Protection Act 2018 and General Data Protection Regulation (2018) legislation. The University of Southampton policy on archiving will be followed; the data will be stored for 10 years following the end of the study, after which time it will be disposed of securely. Following completion of the trial, a cleaned anonymised data set will be shared on request.

### Patient and public involvement

Patient representatives have been involved with the SupportBack trials from the outset. The idea for the trials and their subsequent design was informed by the local branch of the national charity BackCare. From this group, LL joined the research team and contributed to funding applications for both feasibility and main trials. SupportBack 2 has a panel of three patient and public involvement (PPI) representatives who are part of the trial management group, advising on patient facing materials and contributing to discussions of trial related issues as they arise. PPI will pay a key role in dissemination of trial findings and interpretation of qualitative data.

### Ethics and dissemination

The SupportBack 2 trial has received full ethical approval form a local review board (REC Ref: 18/SC/0388). All potentially eligible patients receive a patient information sheet. This information emphasises that participation in the trial is voluntary and that the participant may withdraw from the trial at any time for any reason. The participants are given the opportunity to ask any questions that may arise by speaking with the trial team and time to consider the information fully prior to agreeing to participate.

The findings of this trial will be published in peer-reviewed journals and presented at international conferences. We will develop press releases in order to disseminate the findings to the general public, and work closely with our PPI collaborators to ensure dissemination to patient and other special interest groups. A summary of the findings will be sent to all included general practices and those patients that request this information. If the intervention is shown to be effective, we will work with developers to rapidly develop a version for widescale dissemination and implementation.

## Supplementary Material

Reviewer comments

Author's manuscript
