# Supplementary material for: Supporting self-management of low back pain with an internet intervention in primary care: a protocol for a randomised controlled trial of clinical and cost-effectiveness (SupportBack 2)
Source: BMJ Open. 2020 Aug 20;10(8):e040543. doi: 10.1136/bmjopen-2020-040543 (PMC7440707; doi:10.1136/bmjopen-2020-040543)
Supplement: Author's manuscript [file bmjopen-2020-040543.draft_revisions.pdf]

BMJ Open is committed to open peer review. As part of this commitment we make the peer review history of every article we publish publicly available.

When an article is published we post the peer reviewers' comments and the authors' responses online. We also post the versions of the paper that were used during peer review. These are the versions that the peer review comments apply to.

The versions of the paper that follow are the versions that were submitted during the peer review process. They are not the versions of record or the final published versions. They should not be cited or distributed as the published version of this manuscript.

BMJ Open is an open access journal and the full, final, typeset and author-corrected version of record of the manuscript is available on our site with no access controls, subscription charges or pay-per-view fees (http://bmjopen.bmj.com).

If you have any questions on BMJ Open's open peer review process please email info.bmjopen@bmj.com

BMJ Open

Supporting self-management of low back pain with an internet intervention in primary care: A protocol for a randomised controlled trial of clinical and cost-effectiveness (SupportBack 2)

Journal:	*BMJ Open*
Manuscript ID	bmjopen-2020-040543
Article Type:	Protocol
Date Submitted by the Author:	15-May-2020

Complete List of Authors:	Geraghty, Adam; University of Southampton, Primary Care and Population Sciences<br>Roberts, Lisa; University of Southampton, Health Sciences<br>Hill, Jonathan; Keele University, Institute of primary care and health sciences<br>Foster, Nadine; Keele University, Arthritis Research UK Primary Care Centre<br>Yardley, Lucy ; University of Oxford, Nuffield Department of Primary Health Care Sciences<br>Hay, Elaine; Arthritis Research Primary Care Centre<br>Stuart, Beth; University of Southampton, Primary Care and Population Science<br>Turner, David; University of East Anglia, Public Health and Primary Care<br>Griffiths, Gareth; The University of Southampton<br>Webley, Frances; University of Southampton, Southampton Clinical Trials Unit<br>Durcan, Lorraine; University of Southampton, Southampton CTU<br>Morgan, Alannah; University of Southampton, Southampton Clinical Trials Unit<br>hughes, Stephanie; University of Southampton, Primary Care and Population Sciences<br>Bathers, Sarah ; Keele University, Keele Clinical Trials Unit<br>Butler-Walley, Stephanie; Keele University, Keele Clinical Trials Unit<br>Wathall, Simon; Keele University, Institute of Primary Care & Health Sciences<br>Mansell, Gemma ; Aston University, Psychology<br>Leigh, Linda<br>Little, Paul; University of Southampton, Primary Care and Population Science;

Keywords:	Back pain < ORTHOPAEDIC & TRAUMA SURGERY, PRIMARY CARE, REHABILITATION MEDICINE

For peer review only - http://bmjopen.bmj.com/site/about/guidelines.xhtml

I, the Submitting Author has the right to grant and does grant on behalf of all authors of the Work (as defined in the below author licence), an exclusive licence and/or a non-exclusive licence for contributions from authors who are: i) UK Crown employees; ii) where BMJ has agreed a CC-BY licence shall apply, and/or iii) in accordance with the terms applicable for US Federal Government officers or employees acting as part of their official duties; on a worldwide, perpetual, irrevocable, royalty-free basis to BMJ Publishing Group Ltd ("BMJ") its licensees and where the relevant Journal is co-owned by BMJ to the co-owners of the Journal, to publish the Work in this journal and any other BMJ products and to exploit all rights, as set out in our licence.

The Submitting Author accepts and understands that any supply made under these terms is made by BMJ to the Submitting Author unless you are acting as an employee on behalf of your employer or a postgraduate student of an affiliated institution which is paying any applicable article publishing charge ("APC") for Open Access articles. Where the Submitting Author wishes to make the Work available on an Open Access basis (and intends to pay the relevant APC), the terms of reuse of such Open Access shall be governed by a Creative Commons licence – details of these licences and which Creative Commons licence will apply to this Work are set out in our licence referred to above.

Other than as permitted in any relevant BMJ Author's Self Archiving Policies, I confirm this Work has not been accepted for publication elsewhere, is not being considered for publication elsewhere and does not duplicate material already published. I confirm all authors consent to publication of this Work and authorise the granting of this licence.

For peer review only

Supporting self-management of low back pain with an internet intervention in primary care: A protocol for a randomised controlled trial of clinical and cost-effectiveness (SupportBack 2)

Adam W A Geraghty[1] *, Lisa C Roberts[2], Jonathan Hill[3], Nadine E Foster[3], Lucy Yardley[4], Elaine M Hay[3], Beth Stuart[1], David A Turner[5], Gareth Griffiths[6], Frances Webley[6], Lorraine Durcan[6], Alannah Morgan[6], Stephanie Hughes[1], Sarah Bathers[7], Stephanie Butler-Walley[7], Simon Wathall[7], Gemma Mansell[8], Linda Leigh[7], Paul Little[1].

[1] Primary Care, Population Sciences and Medical Education, University of Southampton, United Kingdom.

[2] School of Health Sciences, University of Southampton, United Kingdom & University Hospital Southampton NHS Foundation Trust, United Kingdom.

[3] Primary Care Centre Versus Arthritis, School of Primary, Community and Social Care, Keele University

[4] School of Psychological Science, University of Bristol and Department of Psychology, University of Southampton, United Kingdom.

[5] Norwich Medical School, University of East Anglia, United Kingdom.

[6] Southampton Clinical Trials Unit, University of Southampton, United Kingdom.

[7] Keele Clinical Trials Unit, School of Primary, Community and Social Care, Keele University, United Kingdom.

[8] Patient and Public Involvement Representative, Southampton, United Kingdom.

[9] School of Life and Health Sciences, Aston University, Birmingham, United Kingdom.

For peer review only

*Correspondence concerning this article should be addressed to Adam W A Geraghty, Primary Care and Population Sciences, Aldermoor Health Centre, Aldermoor Close, Southampton, Hampshire, SO16 5ST. Email: A.W.Geraghty@soton.ac.uk. Tel: (+44) 2380241051

Word Count: 5289

For peer review only

ABSTRACT

Introduction

Self-management and remaining physically active are first-line recommendations for the care of patients with low back pain (LBP). With a life-time prevalence of up to 85%, novel approaches to support behavioural self-management are needed. Internet interventions may provide accessible support for self-management of LBP in primary care. The aim of this randomised controlled trial is to determine the clinical and cost-effectiveness of the 'SupportBack' internet intervention, with or without physiotherapist telephone support in reducing LBP-related disability in primary care patients.

Methods and analysis

A three parallel arm, multi-centre randomised controlled trial will compare three arms: 1) usual primary care for LBP; 2) usual primary care for LBP and an internet intervention; 3) usual primary care for LBP and an internet intervention with additional physiotherapist telephone support. Patients with current LBP and no indicators of serious spinal pathology are identified and invited via general practice list searches and mailouts or opportunistic recruitment following LBP consultations. Participants undergo a secondary screen for possible serious spinal pathology and are then asked to complete baseline measures online after which they are randomised to an intervention arm. Follow-ups occur at six weeks, three, six and 12 months. The primary outcome is physical function (using the Roland and Morris Disability Questionnaire] over 12-months [repeated measures design). Secondary outcomes include pain intensity, troublesome days in pain over the last month, pain self-efficacy, catastrophising, kinesophobia, health-related quality of life and cost-related measures for a full health economic analysis. A full mixed methods process evaluation will be conducted.

For peer review only

Ethics and dissemination

This trial has been approved by an NHS Research Ethics Committee (REC Ref: 18/SC/0388). Results will be disseminated through peer-reviewed journals, conferences, communication with practices and patient groups. Patient representatives will support the implementation of our full dissemination strategy.

Trial registration number: ISRCTN reference 14736486 (pre-results)

Key words: Low Back Pain; Internet Intervention; Primary Care; Randomised Controlled Trial

For peer review only

STRENGHTS AND LIMITATIONS

- The SupportBack 2 trial is a large multi-centre randomised trial that will determine the additional benefit, over usual primary care, of an internet-based approach that supports self-management of patients with LBP in UK primary care.
- The trial is designed to investigate the effectiveness of an internet intervention in addition to usual primary care, both with and without telephone physiotherapist support.
- A full mixed methods process evaluation will be carried out to inform a logic model and 'theory of change' for the interventions.
- Inclusion is limited to those with LBP who have access to the internet and are able to communicate in English without assistance.

For peer review only

INTRODUCTION

Low Back Pain (LBP) has a life-time prevalence of up to 85% [1] and is the greatest single cause of years lived with disability globally. [2] LBP is primarily managed in primary care, [3] where first-line recommendations are to self-manage and remain physically active. [4] Supporting effective behavioural self-management of LBP is increasingly important; the most recent guidelines place less emphasis on pharmacological and surgical treatments. [5] General practitioners are unlikely to have the training or the capacity to support behavioural self-management, and access to specialist musculoskeletal (MSK) services can be variable. [6] New roles such as First Contact Physiotherapists in general practice are emerging, but implementation is at an early stage. [7] Internet interventions may offer a route to rapidly scalable behavioural support for patients with LBP, however their effectiveness in UK primary care needs to be determined.

Internet interventions are typically automated, interactive, tailored interventions that make use of multimedia formats to deliver behaviour change strategies online. [8] Internet interventions are one form of a broader category of digital interventions that draw on digital technologies including the internet, mobile devices and activity sensors. [9] A recent systematic review of digital interventions for LBP highlighted substantial heterogeneity in intervention delivery, duration and outcomes, making it difficult to draw conclusions regarding effectiveness. [10] Since the publication of this review there has been a focus on mobile apps for LBP: a German study has shown that a mobile app delivering multidisciplinary self-management support for patients with LBP recruited via online advertising significantly reduced pain at 12 week follow-up, compared to a 6 week course of exercise delivered by physiotherapists plus online education. [11] An ongoing European programme of work seeks to determine the effectiveness of a mobile app-based digital decision support self-management programme (selfBACK) for patients recently consulting in primary care for LBP. [12]

For peer review only - http://bmjopen.bmj.com/site/about/guidelines.xhtml

Whilst mobile apps show potential, internet interventions likely have an accessibility advantage; they can be accessed from any device with an internet connection (e.g. desktop, laptop, tablet, mobile phone).

SupportBack is an internet intervention designed to support patients to self-manage their LBP following consultation in primary care. [13, 14] It was developed using evidence and theory in combination with the person-based approach, where systematic qualitative research is integrated throughout development. [15, 16] SupportBack is designed to be as accessible as possible, both in presentation style and in target; it can be used by people with both acute and persistent LBP. SupportBack has been developed to be used in addition to usual care, either as a stand-alone internet intervention or in combination with physiotherapist telephone support. A randomised controlled feasibility trial demonstrated both the feasibility of trial procedures and the effective delivery of the intervention and telephone support. [14] In a nested qualitative study within the feasibility trial, Geraghty et al. [15] found that patients were broadly positive about the intervention; they suggested that it provided reassurance whilst supporting becoming more physically active as a primary pain management strategy.

The aim of the present full randomised controlled trial is to determine the clinical and cost-effectiveness of the SupportBack internet intervention, delivered in addition to usual care with and without physiotherapist telephone support, in reducing LBP-related physical disability in UK primary care.

METHODS

Design

A three parallel arm (1:1:1), multicentre randomised controlled trial is being conducted to determine the clinical and cost-effectiveness of an internet intervention for patients

For peer review only - http://bmjopen.bmj.com/site/about/guidelines.xhtml

with LBP in primary care. Participants will be followed up at six weeks, three, six and 12 months.

Study setting

The trial is being carried out with patients from 140-180 general practices across the UK. Patients access the intervention through their own devices with internet access (e.g. a desktop, laptop, tablet, mobile phone) at a location that is convenient for them (e.g. at home or at work). If allocated to receive telephone physiotherapist support, this support is delivered wherever is convenient for the patient. A list of patient identification centres is available from the trial team on request.

Eligibility criteria

Inclusion criteria:

- Aged 18 and above
- Current low back pain (have experienced pain in the last week) with or without sciatica
- Access to the internet and an active email address
- Ability to read/understand English without assistance
- Ability to provide informed consent

Exclusion criteria:

- Signs and symptoms in a patient with LBP that indicate potential serious spinal pathology such as infection, malignancy, fracture, inflammatory back pain, progressive neurology and/or cauda equina.
- Have had spinal surgery in the past six months
- Pregnancy
- Taken part in the prior SupportBack feasibility study

For peer review only

Identification, recruitment and screening

Two recruiting centres, Southampton and Keele [each with a team of telephone support physiotherapists] are working with National Institute for Health Research (NIHR) Clinical Research Networks (CRNs) to facilitate the recruitment of general practices. Potentially eligible participants will be identified in one of two ways:

1) Patients who have consulted with LBP in the last two months will be identified by general practice staff from computerised records of consultations. Practices will be asked to repeat the searches approximately three times, or until the target number of patients per practice has been reached. Resulting lists of patients identified by the search will be screened by a practice GP who will rule out patients based on aspects of the eligibility criteria that can be determined from patient notes.

2) During a patient consultation and on entering a relevant diagnostic or symptom Read code into the patient electronic medical record, GPs will be prompted about the trial and patient eligibility by an automated 'pop-up' screen activated by the Read code. GPs will then screen for eligibility [using the inclusion/exclusion criteria listed] and patients identified as suitable will have their medical record electronically tagged. A download of 'tagged' patients will occur regularly, anticipated to be every two weeks. This method will be used in practices where possible. Participating general practices not implementing the 'pop up' Read code method can identify potential patients during consultation. Having considered eligibility the GP or Nurse Practitioner will provide the patient with an invitation pack.

Patients identified either by a medical records review or general practice consultation are mailed a study pack including an invitation letter from the GP, participant information sheet, reply slip, screening questions and pre-paid envelope. Interested

For peer review only - http://bmjopen.bmj.com/site/about/guidelines.xhtml

patients return the reply slip and screening questions using the pre-paid envelope to the Research Team. Screening consists of two questions regarding current LBP and access to the internet, followed by three safety questions listing symptoms that may indicate serious spinal pathology. Patients who answer 'Yes' to the first two questions, and 'No' to all safety questions, are considered eligible. For those who complete the screening questions and fail safety screening, a physiotherapist contacts the patient to make an appropriate clinical recommendation on hearing a further description of the symptoms. Those who fail the screening are documented on a screening log maintained by the Research Team. All patients considered eligible for the trial are assigned a unique participant identification number and sent a link to the study website, to complete consent, baseline questionnaires and be randomised. Recruitment opened in November 2018 and is expected to close in December 2020, with data collection completing approximately 12 months later in December 2021.

Randomisation, allocation and blinding

The randomisation process for this trial is fully automated. The intervention and data collection software automatically generates the randomisation sequence, and a computer-generated algorithm block randomises participants to the trial groups. Participants are being stratified by trial recruiting centre and level of physical function: a score of less than four on the Roland Morris Disability Questionnaire (RMDQ [17]) is being used to denote a lower level of self-rated physical disability. As the automated software randomises patients, the randomisation sequence is concealed from the trial team. Patients are automatically informed of their group allocation through the internet intervention software. As patients are engaging with a behavioural intervention, they are not blind to allocation. The majority of data will be collected online, or by post. Telephone calls are used to collect primary outcome data where there has been no response to online and postal follow-up. The callers are blind to group allocation. The statisticians conducting the analyses will remain blind to group allocation. The health economist will conduct the majority of analysis blinded to group, however, estimates

For peer review only

of total cost require the addition of costs specific to the provision of the interventions so will become un-blinded at this point. Figure 1 details patient flow through the trial.

[Insert Figure 1 about here]

Interventions

Usual care

Participants randomised to this arm will continue to receive unrestricted usual primary care for LBP. Current NICE recommendations for primary care management of LBP suggest assessment to rule out specific spinal pathology and use of risk stratification tools (e.g. STarT Back [18]). Guidelines with regard to pharmacotherapy recommend NSAIDs at the lowest effective doses for the shortest period of time. Paracetamol and opiates are not recommended for routine use. Care may also include referrals for physiotherapy and psychological interventions such as cognitive behaviour therapy (CBT). Despite guidelines, there is likely to be a variety in what is provided for patients as part of usual care over the trial period from no further contacts, to referrals to physiotherapy and pain clinics. Consequently, health care resource use will be documented and form a central part of our health economic analysis.

Usual care + Internet intervention

Participants randomised to this arm will continue to receive unrestricted usual primary care. In addition, they will have access SupportBack. SupportBack is a multi-session, interactive internet intervention that provides participants with accessible information, advice and tools to support the effective self-management of LBP. The SupportBack intervention [including telephone support] has been extensively described elsewhere. [13, 15] In brief, the central components of the intervention include graded goal setting, self-monitoring, and tailored feedback to encourage physical activity/exercise increases or maintenance. SupportBack also provides educational modules regarding pain and LBP-related topics (relieving pain; flare ups; work; sleep; mood; daily living).

For peer review only - http://bmjopen.bmj.com/site/about/guidelines.xhtml

Throughout the digital material, there is a focus on supporting motivation for behaviour change: Techniques employed include reassuring about consequences of movement, modelling of use of activity as a primary pain management strategy and using automation to deliver positive feedback through reassurance regarding consequences. These techniques were combined with a person-based approach in the development of SupportBack, where the application of such approaches was guided by systematic in-depth qualitative research with patients with LBP. [16]

Practically, patients can access SupportBack from any device with an internet connection from wherever is most convenient for them. SupportBack consists of six sessions, and patients are encouraged to log in and use one session per week. Automated reminders adhere to this schedule. The first session highlights the centrality of physical activity in managing LBP, and supports patients to set goals to either walk more, or engage with a range of gentle back exercises of their choice. Goal options are tailored and are based the extent that patients report their LBP obstructs their day-to-day activities. The further sessions feature self-monitoring and feedback regarding their progress with walking or exercise goals, combined with encouragement from SupportBack to continue. After the first session, patients can unlock one further module per week on topics such as sleep, mood and work. These build into a personal repository, that alongside weekly goals, can be accessed at any time. If engaged with weekly, the tailored, interactive part of the intervention will last six weeks. Following completion of all the sessions, SupportBack converts into static resource where all activities/exercises and modules can be accessed for the duration of the trial.

Usual care + internet intervention + telephone physiotherapist support

Participants randomised to this arm will also continue to receive unrestricted usual primary care, with access to the SupportBack internet intervention. In addition, these participants will also receive up to one hour of physiotherapist support over the telephone (the first call can be up to 30 minutes, with two follow-up calls of up to 15

For peer review only

minutes, over the six-week intervention period). At both centres (Southampton and Keele) support is provided by musculoskeletal physiotherapists working in the NHS.

The objectives of the telephone contact are to encourage the use of the SupportBack intervention, provide reassurance regarding LBP and encourage adherence to physical activity goals. The physiotherapists are asked to closely adhere to a standardised content checklist for each phone call. The checklist follows the CARE approach [19] (Congratulate, Ask, Reassure, Encourage), developed specifically to guide support for digital interventions. Drawing on existing clinical skills, it ensures a general supportive approach and requires minimal training (all support physiotherapists attended a two-hour training session ahead of the trial). Whilst telephone physiotherapists are able to address individual participant concerns, they are asked to avoid additional individualised participant assessment and treatment recommendations beyond the internet intervention content. Physiotherapists complete a checklist for each call. Checklists are returned to the trial coordinating centre, where intervention fidelity will be assessed.

Measures

All measures and time points for collection are listed in Table 1.

Primary outcome

The primary outcome in this trial is LBP-related physical function measured with the Roland Morris Disability Questionnaire. [17] Function forms a central domain in the recommended core outcomes set for LBP trials. [20] The RMDQ is a recommended measure of physical function [21] and is commonly used in primary care LBP trials.

Secondary measures

For peer review only

Demographic data is being collected at baseline including age, sex, educational attainment, marital and occupational status. A range of secondary measures are being collected including pain intensity, [22] number of troublesome days in pain [23] and risk of pain related disability, [24] Pain-related psychological variables are being measured including kinesiophobia [fear of movement], [25] catastrophizing, [26] pain self-efficacy, [27] outcome expectations [28] and symptoms of depression and anxiety. [29] General physical activity is being measured with the Godin Leisure-time Exercise Questionnaire [30] in this trial; the short-form IPAQ [31] was used in the feasibility trial but produced unreliable data. We are also measuring intervention specific physical activity with a single item developed for this trial. Adherence to walking and physical activity goals are being measured with specifically developed items, based closely on measures previously used in a related behavioural trial [32] and difficulties with the intervention are being measured using the Problematic Experiences of Therapy Scale. [33]

To support the health economic analysis health-related quality of life is being measured with the EQ-5D-5L. [34] All resources required to provide the internet intervention and the telephone support will be recorded. Details of NHS resource use will be recorded from general practice notes review. This will include both primary and secondary care contacts and will cover both general health care usage in addition to LBP specific care in the follow-up period. Additionally, LBP specific medication use will be captured. There may also be differences in LBP related services paid for by study participants: for example, complementary or alternative medicine (CAM). Participants may also require time off work. Additionally, there may be under-reporting of LBP-specific resource use from medical records. These resources will be captured by means of a simple questionnaire administered at six and 12 months. The time-off work question and items relating to use of private health care will additionally be asked at baseline. All resources identified will be costed using appropriate local and national data, for example NHS reference costs and Unit Costs of Health and Social Care. Occupational

For peer review only - http://bmjopen.bmj.com/site/about/guidelines.xhtml

status is being measured with a brief questionnaire developed for this trial.

The internet intervention software automatically collects data on number of logins, page and module views and time spent in each login. This data will be used to explore adherence and user engagement to the digital component of the intervention.

Table 1. Outcomes and measures used in the trial

**Domain**	**Measure**	**Time point**
Function (Primary outcome)		
LBP-related physical function	Roland Morris Disability Questionnaire (RMDQ) [17]	Baseline, 6 weeks, 3, 6, 12 months. All arms.
Pain		
Pain intensity	Pain index (Numerical rating scales measuring current, average and least pain over the last 2 weeks) [22]	Baseline, 6 weeks, 3, 6, 12 months. All arms.
Pain duration	Time since last pain free month [35]	Baseline. All arms.
Troublesomeness of pain	Troublesome days in pain over the last month [developed from days in pain measure [23]	Baseline, 6 weeks, 3, 6, 12, months. All arms.
Risk of persistent pain-related disability	STarT Back tool [24]	Baseline, 12 months. All arms.
Psychological processes related to pain		
Fear of movement	Tampa Scale for Kinesiophobia (TSK-11) [25]	Baseline, 12 months.<br>All arms.
Catastrophising/negative orientation towards pain	Pain Catastrophizing Scale (PCS) [26]	Baseline, 12 months follow-up<br>All arms.
Confidence in ability to manage pain	Pain Self-Efficacy Questionnaire (PSEQ) [27]	Baseline, 6 weeks, 3, 6, 12 months. All arms.
Self-efficacy for managing LBP	Single item from Musculoskeletal Health Questionnaire (MSK-HQ) [36]	Baseline, 6 weeks, 3, 6, 12 months. All arms.

For peer review only

Outcome expectation	Expectancy question from Credibility and Expectancy Questionnaire modified for LBP CEQ low back pain modification [28, 37]	Baseline, all arms. following session one of SupportBack Internet intervention arms only.
Mental health	Patient Health Questionnaire-4 [29] depression and anxiety measure	Baseline, 12 months. All arms.
Physical activity/adherence		
General physical activity	Godin leisure-time exercise questionnaire [30]	Baseline, 12 months. All arms
SupportBack-related physical activity	Single item measure developed for the trial	Baseline, 6 weeks, 3, 6, 12 months. All arms.
Adherence to back-specific activity	Item developed for this trial, based on previous behavourial adherence measures [32]	12 months. All arms
Difficulties with intervention recommendations	Problematic Experiences of Therapy Scale (PETS) [33]	12 months. Internet intervention arms only.
Satisfaction and enablement		
Satisfaction with care received for LBP	Single satisfaction item developed for trial	6 weeks. All arms.
Enablement	Patient Enablement Instrument (PEI) [38]	6 weeks, 12 months. All arms.
Health related quality of life, health care resource use, and occupational status		
Health-related Quality of Life	ED-5D Five Level [34]	Baseline, 6 weeks, 3, 6, 12 months. All arms.
Use of over-the-counter (OTC) medication for LBP	Single item measuring self-reported OTC medication usage for LBP	Baseline, 6 months, 12 months. All arms.
Participant borne costs	Participant reported health resource use questionnaire developed for this study	Baseline, 6 months, 12 months. All arms.
NHS health care resource use (specific to back pain, and general)	General practice medical notes review and Participant reported health care resource use questionnaire developed for this trial	Baseline, 6 months, 12 months. All arms.
Occupational impact of LBP	Brief occupational questionnaire developed for this trial.	12 months. All arms.
Use of internet resources		

For peer review only

Use of internet resources	Single item regarding use of internet resources for LBP over trial period.	12 months.<br>All arms.

Sample size

The reported minimally clinical important difference (MCID) between groups for the RMDQ varies. A between group MCID of 2 or 3 points is commonly reported. [23, 39, 40] However, it has been suggested that a difference of 1.5 may still be important, particularly in the context of low intensity interventions. [23] Whether delivered as a stand-alone intervention or coupled with brief telephone support, SupportBack is a low intensity intervention with the potential to be rapidly scalable. Consequently, we considered a between group change of at least 1.5 to be a meaningful difference in this context. For our repeated measures primary outcome, a difference of 1.5 points on the RMDQ over the follow-up period of 12 months, assuming a standard deviation of 5 in line with the feasibility study, [23] gives an effect size of 0.30. Alpha will be set to 0.025 to allow both interventions to be independently compared with the usual care alone arm. Using four repeated measures (six weeks, three, six and 12 months), and assuming a correlation between repeated measures of 0.7 and 90% power, requires 215 participants per arm. Allowing for 20% loss to follow up, this gives a total sample size of 806.

Data collection and management

Data are primarily being collected online. The LifeGuide intervention and data system collects consent, baseline data including demographics and follow-up data across the four time points (six weeks, three, six and 12 months). When first sent a link to the system following screening, if patients do not log on within a week, they are emailed to check that they received the link and advised to look in their spam mail. If there is no response, one telephone call is attempted by the research team.

For peer review only - http://bmjopen.bmj.com/site/about/guidelines.xhtml

With regard to follow-up protocol, where there is no response to the online follow-up questionnaire emails, two reminder emails and text messages will be sent. Following continued non-response, a paper questionnaire pack with a pre-paid envelope will be sent one week after the last email/text reminder. If the paper questionnaires are not returned within two weeks of being sent, a blinded research assistant will call the participant to complete the primary outcome measure (RMDQ), quality of life questionnaire (EQ-5D-5L) and pain severity. If the participant is happy to continue, further measures from the questionnaire battery at the respective follow-up point will be collected in this manner. The full follow-up protocol with the telephone calls will be implemented at six week and 12 month follow-up points. These two follow-up points are considered most important, capturing initial and long-term response. Calling at all time points may lead to increased dropout at later time points. Follow-up at three and six months will include all the above steps except for the phone calls. All participants will receive a £5 voucher when asked to complete questionnaires at the more distant time-points of six and 12 months. Examples of data collection forms can be provided by the trial team on request.

Statistical methods

Quantitative analysis will begin following cleaning and inspection of the data. Descriptive analysis will be conducted to determine outliers and distributions of the data. Where necessary, if data are not normally distributed, transformations will be applied or another appropriate distribution used. The primary analysis for the RMDQ score will be performed using a multilevel mixed model (MLMM) framework with observations at six weeks, three, six and 12 months (level one) nested within participants (level two). Results will be reported adjusting for baseline severity in function, stratification factors and any pre-specified confounders. The model will use all the observed data and makes the assumption that missing RMDQ scores are missing at random given the observed data.

For peer review only

As there may not be a constant treatment effect over time, a treatment/time interaction will be modelled and included if significant (at the 5% level), with time treated as a random effect. An unstructured covariance matrix will be used.

Analysis of secondary outcomes will also be conducted using linear regression for continuous outcomes and logistic regression for dichotomous outcomes, again controlling for baseline symptom severity, stratification factors and any potential confounders. The structure and pattern of missing data will be examined, if appropriate, and a sensitivity analysis based on data imputed using a multiple imputation model presented. Data will be analysed on an intention-to-treat basis [they will be analysed as randomised]. We will also undertake a complier-average causal effect (CACE) analysis, [41] which compares compliant participants in the intervention group, with those in the control group whose characteristics are similar enough to the intervention group compliers to suggest they too would have complied with the intervention, given the opportunity to do so. Compliance for these analyses in the intervention arm will be defined as completing at least Session 1 of the internet intervention. Session 1 contains the central rationale for the intervention; that physical activity is primary in the management of LBP and provides instructions and advice on goal setting. The latter sessions follow a similar format to the first introductory module. With regard to the physiotherapist telephone support arm, we consider per protocol to be receiving at least two of the three planned phone calls. The telephone element is designed to be pragmatic with the necessary flexibility to fit patients' requirements. However, receiving at least two of three calls indicates that support was delivered over time; an important aspect in the design and integration with the internet intervention.

It is not anticipated that there will be significant practice level [cluster] effects but this assumption will be tested by comparing a fixed effect model to a random effects model. If there are significant practice level effects then, the model will include a random effect for practice [random intercept] and participant [random intercept and slope on time] to

For peer review only - http://bmjopen.bmj.com/site/about/guidelines.xhtml

allow for between participant and practice differences at baseline and between participant differences in the rate of change over time (if significant at the 5% level), and fixed effects for baseline covariates.

No interim analyses are planned. Full details of the analyses to be undertaken will be set out in the Statistical Analysis Plan and approved by the Trial Steering Committee (TSC). Our full statistical analysis plan will be published on the trial website in due course (https://www.southampton.ac.uk/medicine/academic_units/projects/supportback2.page).

Cost-effectiveness analysis

A 'within trial' economic analysis will be conducted alongside the RCT to estimate the incremental cost-effectiveness of the SupportBack 2 interventions compared to usual care. The base case perspective will be that of the NHS, but other resources relevant to LBP will be collected to enable additional analysis from a societal perspective.

The main outcome measure in the economic evaluation will be the quality adjusted life year (QALY), obtained from the EQ-5D-5L instrument using the published UK value set. In addition, a cost-effectiveness analysis will be carried out using the study primary outcome measure, i.e. the cost per point change in back-related physical function measured using the RMDQ will be estimated. Both costs and effects will be estimated using multiple regression, to allow for potential confounders, such as baseline scores for EQ-5D-5L and RMDQ. Standard practice will be followed to calculate incremental cost-effectiveness ratios (ICERs), and present ICER(s) where any one option has both higher costs and increased effects compared to another. ICERs will show incremental cost per QALY or incremental cost per point improvement in RMDQ. Bootstrapping will be used to calculate cost-effectiveness acceptability curves (CEACs). These will illustrate the effect of uncertainty on study results. Major

For peer review only - http://bmjopen.bmj.com/site/about/guidelines.xhtml

assumptions made in the analysis will be tested by means of sensitivity analysis. In particular, assumptions made during the costing of the intervention such as the number of individuals who will be using the website will be explored. Similar methods to the main clinical analysis will be used to handle missing data, i.e., analysis of patterns of missing data with multiple imputation methods employed if deemed appropriate. The proposed health economics analysis will be detailed in a health economics analysis plan (HEAP) which will be completed before analysis commences. The HEAP will be circulated for comment prior to the health economics analysis. Any digressions from the HEAP will be documented and justified in the final health economics report.

Process evaluation

A process evaluation will be carried out following Medical Research Council (MRC) guidelines on process evaluations of complex interventions. [42] In order to provide a detailed understanding of the SupportBack intervention three aspects will be examined: Implementation, mechanisms of impact (mediators) and context (moderators). A mixed methods approach will be used to explore these elements.

Implementation

Quantitative data describing trial implementation will be presented including number of practices recruited, patient eligibility [including reasons for declined participation where possible, and analysis of screen failures] and recruitment rates. The number of withdrawals from the trial per arm will be presented, along with numbers/percentages of dropouts from the intervention who do not respond to follow-up. Use of the internet intervention will be described by presenting automated data collected on number of logins and modules accessed for both the internet intervention and the intervention plus telephone physiotherapist support arm. With regard to the internet intervention plus telephone physiotherapist support arm, the number of support calls successfully made (and attempts contact the patient), along with the mean number per

For peer review only

participant in this arm will be described.

Qualitative interviews will be conducted with up to 45 trial participants following the three, six and 12-month follow-up points. Different participants will be interviewed at each time point, enabling us to explore how time since accessing the tailored weekly component of the intervention effects how suggestions are used and implemented in daily life. Interviews will also be conducted with the trial physiotherapists. Participants will be purposively sampled to ensure diversity in terms of age, sex and symptom severity (physical function, pain intensity and duration). Participants will also be sampled based on high and low usage of the internet intervention and high and low engagement with the telephone physiotherapist support. For participants, questions will focus on their experience of using the intervention, including telephone physiotherapist support and usual care. Interviews with the trial support physiotherapists will be designed to explore their experience of delivering the intervention, with a particular focus on barriers and facilitators, and determinants of successful exchanges.

Mechanisms of impact

A logic model of proposed mechanisms affecting LBP-related physical disability and pain outcomes for the SupportBack intervention has been developed (see [14]). This model will be used as the basis of both quantitative and qualitative exploration of mechanisms. Quantitative analyses will focus on psychological and behavioural mechanisms influencing outcome following use of the interventions including expectancy, self-efficacy to manage LBP, physical activity, self-reported goal setting across the intervention and objective measures of intervention use (sessions completed, use of additional modules, e.g. mood, sleep etc.). In order to explore whether two core mechanisms' [mediating variables] contribution to outcome is unique to the internet intervention arms, brief single items capturing self-efficacy (SE) and physical activity (PA) are being measured in all three arms (including usual care). SE and PA are being

For peer review only - http://bmjopen.bmj.com/site/about/guidelines.xhtml

measured at baseline and in the outcome questionnaire sets at six weeks, three, six, and 12 months. Mediation analysis will be used to explore relationships between mediating variables and LBP-related physical function and pain intensity across the 12-month follow-up period. We will also explore the potential of multilevel modelling to examine mediating variables association with the outcome over time. [43] Appropriate checks of the assumptions of causal modelling, such as exchangeability (no confounding), consistency, effect modification and temporality will also be carried out. [44, 45]

Questions will be included in the qualitative interviews focusing on participants' perceptions of how use of the SupportBack intervention and/or telephone support affected their LBP. This will enable the inductive exploration of participants' views and triangulation of qualitatively derived theory on mechanism with our quantitative analysis. Similar questions will also be explored in the usual care arm, focusing on how elements of their usual care may have led to improvements in their LBP.

Context

The relationship between elements of participants' context [moderators] and the effect of the interventions across the 12-month follow-up period will be explored. This will include variables such as LBP severity and duration at baseline, age, educational level and occupation status. Following the analysis of mechanisms, correlations and multiple regression (linear and logistic) will be used to explore relationships between moderating variables and LBP-related physical function and pain intensity. Qualitatively, the above aspects of participants' context, including their own descriptions of their LBP history, will feed into analysis when exploring themes regarding participants use of the intervention and their perceptions of benefit. [46]

Qualitative analysis

For peer review only - http://bmjopen.bmj.com/site/about/guidelines.xhtml

For peer review only

Interview data collected regarding implementation, mechanisms and context will be transcribed verbatim, coded and analysed using an inductive thematic analytic approach. [47, 48] This will ensure participants' qualitative data are not constrained by the direction of a particular theoretical model, and enable novel insights from qualitative work to be added into the theory-driven logic model. A key aspect of the qualitative analysis will entail exploring differences in accounts at different time points. This will enable us explore how time since the tailored weekly sessions impacts on the process of self-management.

Data monitoring and confidentiality

The SupportBack 2 trial has a data monitoring and ethics committee (DMEC) composed of a statistician (chair) and two academic clinicians (Professor in Primary Care Research and Professor of Physiotherapy respectively). The DMEC reports to the Trial Steering Committee (TSC) and is fully independent from the trial Sponsor with no competing interests. Interim descriptive analyses are prepared for the DMEC. The DMEC charter can be obtained from the research team on request.

All Serious Adverse Events (SAEs) are reported to the lead Clinical Trial Unit (CTU). The assessment of seriousness will be made by the participants GP or delegate. Assessment of causality will be made by the GP or delegate, and related or unrelated status will be determined. As the SupportBack intervention provides reassurance and encourages gentle activity within the participants' own limits, there are no 'expected' SAEs documented.

All patient data are being kept in strict confidence and managed in accordance with the Data Protection Act 2018 and General Data Protection Regulation (GDPR 2018) legislation. The University of Southampton policy on archiving will be followed; the data will be stored for 10 years following the end of the study, after which time it will

For peer review only - http://bmjopen.bmj.com/site/about/guidelines.xhtml

be disposed of securely. Following completion of the trial, a cleaned anonymised data set will be shared on request.

Patient and public involvement (PPI)

Patient representatives have been involved with the SupportBack trials from the outset. The idea for the trials and their subsequent design was informed by the local branch of the national charity BackCare. From this group, LL joined the research team and contributed to funding applications for both feasibility and main trials. SupportBack 2 has a panel of three PPI representatives who are part of the trial management group (TMG), advising on patient facing materials and contributing to discussions of trial related issues as they arise. PPI will pay a key role in dissemination of trial findings and interpretation of qualitative data.

Ethics and dissemination

The SupportBack 2 trial has received full ethical approval form a local review board (REC Ref: 18/SC/0388). All potentially eligible patients receive a patient information sheet. This information emphasises that participation in the trial is voluntary and that the participant may withdraw from the trial at any time for any reason. The participants are given the opportunity to ask any questions that may arise by speaking with the trial team and time to consider the information fully prior to agreeing to participate.

The findings of this trial will be published in peer reviewed journals and presented at international conferences. We will develop press releases in order to disseminate the findings to the general public, and work closely with our PPI collaborators to ensure dissemination to patient and other special interest groups. A summary of the findings will be sent to all included general practices and those patients that request this information. If the intervention is shown to be effective, we will work with developers to rapidly develop a version for widescale dissemination and implementation.

For peer review only - http://bmjopen.bmj.com/site/about/guidelines.xhtml
For peer review only

Author contributions

AWAG, PL, LCR, NEF, JCH, LY, DAT, EMH and GG designed the trial and secured funding. AWAG led the development of the internet intervention with LY and LCR, with further input from NEF, JCH, EMH, PL and LL. LY, AWAG, LCR, JCH, NEF, developed the physiotherapy telephone support package. BS wrote the statistical analysis plan reported in the protocol, and DAT wrote the health economic plan. AWAG drafted the manuscript with input from all authors. AWAG is the guarantor.

Acknowledgements

There are a number of individuals we would like to thank for their ongoing support for this trial: Alastair Mobley at the CRN West Midlands for help with recruitment; Anna Thornhill, Emma Mcloughlin, Jacqui Tomkins, Zoe Oram, Caroline Harwood, for screening patients; Jessica White, Julie Thorpe, Kathryn Palmer, Sarah Olivant, Michael Gillingham, Katrina Humphreys, Lucy Huckfield, Tina Hadley-Barrows, and Yvonne Rimmer for providing telephone support; Dr Sarah Williams and Solent NHS Trust for supporting key setup and trial processes. SupportBack was developed using LifeGuide software and methodologies, which was partly funded by the NIHR Southampton Biomedical Research Centre (BRC).

Funding statement

This work was supported by the National Institute of Health Research (NIHR) Health Technology Assessment (HTA) programme, grant number 16/111/78. This protocol describes independent research funded by the NIHR. The views expressed are those of the author[s] and not necessarily those of the NHS, the NIHR or the Department of

For peer review only

Health and Social Care. NEF was funded by an NIHR Research Professorship (NIHR-RP-011-015), LCR was funded by a NIHR Senior Clinical Lectureship (round 3) and NEF, EMH and PL are NIHR Senior Investigators. LY is an NIHR Senior Investigator and her research programme is partly supported by NIHR Applied Research Collaboration (ARC)-West, NIHR Health Protection Research Unit (HPRU) for Behavioural Science and Evaluation, and the NIHR Southampton Biomedical Research Centre (BRC)

For peer review only

Competing interests statement

Authors declare no competing interests.

Access to data

Data will be available to access from the authors on reasonable request.

REFERENCES

1. Andersson GB. Epidemiological features of chronic low-back pain. Lancet. 1999;354:581-585.
2. Hoy D, March L, Brooks P et al. The global burden of low back pain: estimates from the Global Burden of Disease 2010 study. Ann Rheum Dis. 2014;73:968-974.
3. Koes BW, van Tulder M, Lin CW, Macedo LG, McAuley J, Maher C. An updated overview of clinical guidelines for the management of non-specific low back pain in primary care. Eur Spine J. 2010;19:2075-2094.
4. National Institute for Health and Care Excellence. Low back pain and sciatica in over 16s: assessment and management. London: NICE; 2016

For peer review only - http://bmjopen.bmj.com/site/about/guidelines.xhtml

5. Foster NE, Anema JR, Cherkin D et al. Prevention and treatment of low back pain: evidence, challenges, and promising directions. Lancet. 2018;391:2368-2383.
6. Salisbury C, Foster NE, Hopper C et al. A pragmatic randomised controlled trial of the effectiveness and cost-effectiveness of 'PhysioDirect' telephone assessment and advice services for physiotherapy. Health Technol Assess. 2013;17:1-157, v.
7. Improvement NHSEANHS. Elective care high impact interventions: First contact practitioner for MSK services. 20191-20.
8. Ritterband LM, Gonder-Frederick LA, Cox DJ, Clifton AD, West RW, Borowitz SM. Internet interventions: In review, in use, and into the future. Prof Psychol Res Pr. 2003;34:527.
9. Yardley L, Spring BJ, Riper H et al. Understanding and promoting effective engagement with digital behavior change interventions. Am J Prev Med. 2016;51:833-842.
10. Nicholl BI, Sandal LF, Stochkendahl MJ et al. Digital Support Interventions for the Self-Management of Low Back Pain: A Systematic Review. J Med Internet Res. 2017;19:e179.
11. Toelle TR, Utpadel-Fischler DA, Haas KK, Priebe JA. App-based multidisciplinary back pain treatment versus combined physiotherapy plus online education: a randomized controlled trial. NPJ Digit Med. 2019;2:34.
12. Sandal LF, Stochkendahl MJ, Svendsen MJ et al. An App-Delivered Self-Management Program for People With Low Back Pain: Protocol for the selfBACK Randomized Controlled Trial. JMIR Res Protoc. 2019;8:e14720.
13. Geraghty AWA, Stanford R, Little P et al. Using an internet intervention to support self-management of low back pain in primary care: protocol for a randomised controlled feasibility trial (SupportBack). BMJ Open. 2015;5:e009524.
14. Geraghty AWA, Stanford S, Stuart B et al. Using an internet intervention to support self-management of low back pain in primary care: Findings from a randomised controlled feasibility trial (SupportBack). BMJ Open. 2018;8:e016768.

For peer review only

15. Geraghty AWA, Roberts LC, Stanford R et al. Exploring Patients' Experiences of Internet-Based Self-Management Support for Low Back Pain in Primary Care. Pain Med. 2019

16. Yardley L, Morrison L, Bradbury K, Muller I. The person-based approach to intervention development: application to digital health-related behavior change interventions. J Med Internet Res. 2015;17:e30.

17. Roland M, Fairbank J. The Roland-Morris Disability Questionnaire and the Oswestry Disability Questionnaire. Spine [Phila Pa 1976]. 2000;25:3115-3124.

18. Hill JC, Dunn KM, Main CJ, Hay EM. Subgrouping low back pain: a comparison of the STarT Back Tool with the Orebro Musculoskeletal Pain Screening Questionnaire. Eur J Pain. 2010;14:83-89.

19. Bradbury K, Morton K, Band R et al. Understanding how primary care practitioners perceive an online intervention for the management of hypertension. BMC Med Inform Decis Mak. 2017;17:5.

20. Chiarotto A, Deyo RA, Terwee CB et al. Core outcome domains for clinical trials in non-specific low back pain. Eur Spine J. 2015;24:1127-1142.

21. Chiarotto A, Boers M, Deyo RA et al. Core outcome measurement instruments for clinical trials in nonspecific low back pain. Pain. 2018;159:481-495.

22. Hill JC, Dunn KM, Lewis M et al. A primary care back pain screening tool: identifying patient subgroups for initial treatment. Arthritis Rheum. 2008;59:632-641.

23. Little P, Lewith G, Webley F et al. Randomised controlled trial of Alexander technique lessons, exercise, and massage [ATEAM] for chronic and recurrent back pain. BMJ. 2008;337:a884.

24. Hill JC, Whitehurst DG, Lewis M et al. Comparison of stratified primary care management for low back pain with current best practice [STarT Back]: a randomised controlled trial. Lancet. 2011;378:1560-1571.

25. Tkachuk GA, Harris CA. Psychometric properties of the Tampa Scale for Kinesiophobia-11 [TSK-11]. J Pain. 2012;13:970-977.

For peer review only

26. Picavet HS, Vlaeyen JW, Schouten JS. Pain catastrophizing and kinesiophobia: predictors of chronic low back pain. Am J Epidemiol. 2002;156:1028-1034.

27. Nicholas MK. The pain self-efficacy questionnaire: Taking pain into account. Eur J Pain. 2007;11:153-163.

28. Devilly GJ, Borkovec TD. Psychometric properties of the credibility/expectancy questionnaire. J Behav Ther Exp Psychiatry. 2000;31:73-86.

29. Kroenke K, Spitzer RL, Williams JB, Löwe B. An ultra-brief screening scale for anxiety and depression: the PHQ-4. Psychosomatics. 2009;50:613-621.

30. Godin G, Shephard RJ. A simple method to assess exercise behavior in the community. Can J Appl Sport Sci. 1985;10:141-146.

31. Craig CL, Marshall AL, Sjostrom M et al. International physical activity questionnaire: 12-country reliability and validity. Med Sci Sports Exerc. 2003;35:1381-1395.

32. Yardley L, Kirby S. Evaluation of booklet-based self-management of symptoms in Meniere disease: a randomized controlled trial. Psychosom Med. 2006;68:762-769.

33. Kirby S, Donovan-Hall M, Yardley L. Measuring barriers to adherence: validation of the problematic experiences of therapy scale. Disabil Rehabil. 2014

34. Oppe M, Devlin NJ, van Hout B, Krabbe PF, de Charro F. A program of methodological research to arrive at the new international EQ-5D-5L valuation protocol. Value Health. 2014;17:445-453.

35. Dunn KM, Croft PR. The importance of symptom duration in determining prognosis. Pain. 2006;121:126-132.

36. Hill JC, Kang S, Benedetto E et al. Development and initial cohort validation of the Arthritis Research UK Musculoskeletal Health Questionnaire [MSK-HQ] for use across musculoskeletal care pathways. BMJ Open. 2016;6:e012331.

37. Smeets RJ, Beelen S, Goossens ME, Schouten EG, Knottnerus JA, Vlaeyen JW. Treatment expectancy and credibility are associated with the outcome of both physical and cognitive-behavioral treatment in chronic low back pain. Clin J Pain. 2008;24:305-315.

For peer review only

38. Howie JG, Heaney DJ, Maxwell M, Walker JJ. A comparison of a Patient Enablement Instrument [PEI] against two established satisfaction scales as an outcome measure of primary care consultations. Fam Pract. 1998;15:165-171.
39. Bombardier C, Hayden J, Beaton DE. Minimal clinically important difference. Low back pain: outcome measures. J Rheumatol. 2001;28:431-438.
40. UK BEAMTT. United Kingdom back pain exercise and manipulation [UK BEAM] randomised trial: effectiveness of physical treatments for back pain in primary care. BMJ. 2004;329:1377.
41. Knox CR, Lall R, Hansen Z, Lamb SE. Treatment compliance and effectiveness of a cognitive behavioural intervention for low back pain: a complier average causal effect approach to the BeST data set. BMC Musculoskelet Disord. 2014;15:17.
42. Moore GF, Audrey S, Barker M et al. Process evaluation of complex interventions: Medical Research Council guidance. BMJ. 2015;350:h1258.
43. Mansell G, Hill JC, Main CJ, Von Korff M, Van Der Windt D. Mediators of treatment effect in the back in action trial: using latent growth modeling to take change over time into account. Clin J Pain. 2017;33:811.
44. Lee H, Herbert RD, Lamb SE, Moseley AM, McAuley JH. Investigating causal mechanisms in randomised controlled trials. Trials. 2019;20:524.
45. How can we design low back pain intervention studies to better explain the effects of treatment [editorial]. Spine [Phila Pa 1976] 2014;39[5]:E305.
46. Rogers PJ. Using programme theory to evaluate complicated and complex aspects of interventions. Evaluation. 2008;14:29-48.
47. Braun V, Clarke V. Using thematic analysis in psychology. Qual Res Psych. 2006;3:77-101.
48. Joffe H, Yardley L. Content and thematic analysis. In: Marks DF, Yardley L, editors. Research Methods for Clinical and Health Psychology. London: Sage; 2004. p. 56-68.

For peer review only

For peer review only - http://bmjopen.bmj.com/site/about/guidelines.xhtml

For peer review only

Figure 1. Flow through the trial

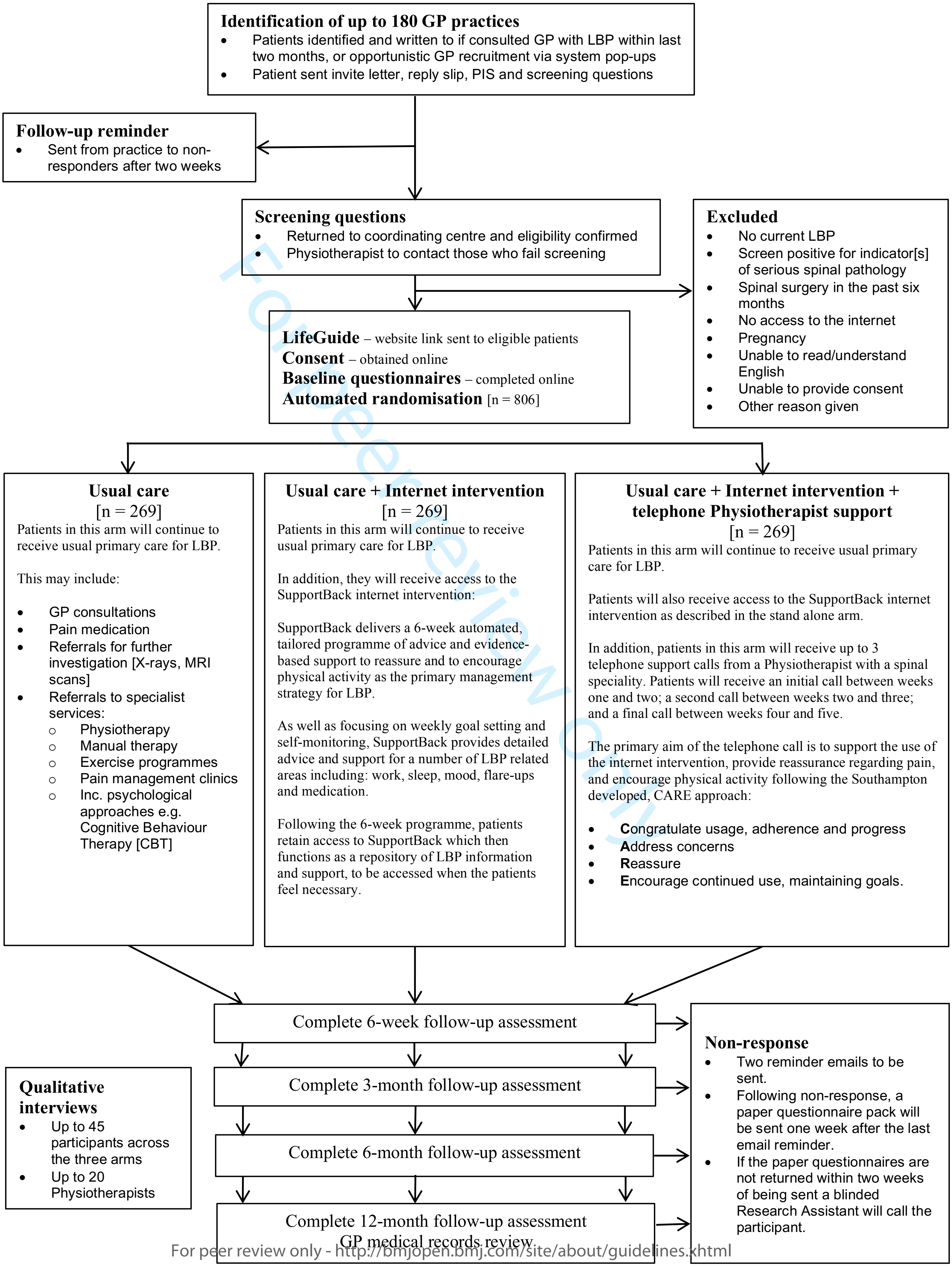

For peer review only - http://bmjopen.bmj.com/site/about/guidelines.xhtml

SPIRIT

STANDARD PROTOCOL ITEMS: RECOMMENDATIONS FOR INTERVENTIONAL TRIALS

SPIRIT 2013 Checklist: Recommended items to address in a clinical trial protocol and related documents*

Section/item	Item No	Description
**Administrative information**		
Title	1	Descriptive title identifying the study design, population, interventions, and, if applicable, trial acronym. **See page 1**
Trial registration	2a	Trial identifier and registry name. If not yet registered, name of intended registry. **See page 2**
	2b	All items from the World Health Organization Trial Registration Data Set. **See page 2, All can be found on ISRCTN register website.**
Protocol version	3	Date and version identifier. **N/A. This can be added if necessary.**
Funding	4	Sources and types of financial, material, and other support. **See page 20-21.**
Roles and responsibilities	5a	Names, affiliations, and roles of protocol contributors. **See page 1 for names and affiliations and page 20 for contributors.**
	5b	Name and contact information for the trial sponsor. **This can be added in necessary.**
	5c	Role of study sponsor and funders, if any, in study design; collection, management, analysis, and interpretation of data; writing of the report; and the decision to submit the report for publication, including whether they will have ultimate authority over any of these activities. **See page 20-21.**
	5d	Composition, roles, and responsibilities of the coordinating centre, steering committee, endpoint adjudication committee, data management team, and other individuals or groups overseeing the trial, if applicable (see Item 21a for data monitoring committee). **See page 18-19.**
**Introduction**		
Background and rationale	6a	Description of research question and justification for undertaking the trial, including summary of relevant studies (published and unpublished) examining benefits and harms for each intervention. **See page 4-5.**
	6b	Explanation for choice of comparators. **See page 4-5.**

For peer review only - http://bmjopen.bmj.com/site/about/guidelines.xhtml

Objectives	7	Specific objectives or hypotheses. **See page 4-5.**
Trial design	8	Description of trial design including type of trial (eg, parallel group, crossover, factorial, single group), allocation ratio, and framework (eg, superiority, equivalence, noninferiority, exploratory). **See page 5.**

Methods: Participants, interventions, and outcomes

Study setting	9	Description of study settings (eg, community clinic, academic hospital) and list of countries where data will be collected. Reference to where list of study sites can be obtained. **See page 5.**
Eligibility criteria	10	Inclusion and exclusion criteria for participants. If applicable, eligibility criteria for study centres and individuals who will perform the interventions (eg, surgeons, psychotherapists). **See page 5-6.**
Interventions	11a	Interventions for each group with sufficient detail to allow replication, including how and when they will be administered. **See page 8-10.**
	11b	Criteria for discontinuing or modifying allocated interventions for a given trial participant (eg, drug dose change in response to harms, participant request, or improving/worsening disease). **N/A**
	11c	Strategies to improve adherence to intervention protocols, and any procedures for monitoring adherence (eg, drug tablet return, laboratory tests). **See page 16-18.**
	11d	Relevant concomitant care and interventions that are permitted or prohibited during the trial. **See page 8-10.**
Outcomes	12	Primary, secondary, and other outcomes, including the specific measurement variable (eg, systolic blood pressure), analysis metric (eg, change from baseline, final value, time to event), method of aggregation (eg, median, proportion), and time point for each outcome. Explanation of the clinical relevance of chosen efficacy and harm outcomes is strongly recommended. **See page 10-12.**
Participant timeline	13	Time schedule of enrolment, interventions (including any run-ins and washouts), assessments, and visits for participants. A schematic diagram is highly recommended (see Figure). **See Figure 1.**
Sample size	14	Estimated number of participants needed to achieve study objectives and how it was determined, including clinical and statistical assumptions supporting any sample size calculations. **See page 13.**
Recruitment	15	Strategies for achieving adequate participant enrolment to reach target sample size. **See page 6-7.**

Methods: Assignment of interventions (for controlled trials)

Allocation:

For peer review only - http://bmjopen.bmj.com/site/about/guidelines.xhtml

Sequence generation	16a	Method of generating the allocation sequence (eg, computer-generated random numbers), and list of any factors for stratification. To reduce predictability of a random sequence, details of any planned restriction (eg, blocking) should be provided in a separate document that is unavailable to those who enrol participants or assign interventions. **See page 7-8.**
Allocation concealment mechanism	16b	Mechanism of implementing the allocation sequence (eg, central telephone; sequentially numbered, opaque, sealed envelopes), describing any steps to conceal the sequence until interventions are assigned. **See page 7-8.**
Implementation	16c	Who will generate the allocation sequence, who will enrol participants, and who will assign participants to interventions. **See page 7-8.**
Blinding (masking)	17a	Who will be blinded after assignment to interventions (eg, trial participants, care providers, outcome assessors, data analysts), and how. **See page 7-8.**
	17b	If blinded, circumstances under which unblinding is permissible, and procedure for revealing a participant's allocated intervention during the trial. **N/A.**
**Methods: Data collection, management, and analysis**		
Data collection methods	18a	Plans for assessment and collection of outcome, baseline, and other trial data, including any related processes to promote data quality (eg, duplicate measurements, training of assessors) and a description of study instruments (eg, questionnaires, laboratory tests) along with their reliability and validity, if known. Reference to where data collection forms can be found, if not in the protocol. **See page 13-14.**
	18b	Plans to promote participant retention and complete follow-up, including list of any outcome data to be collected for participants who discontinue or deviate from intervention protocols. **See page 13-14.**
Data management	19	Plans for data entry, coding, security, and storage, including any related processes to promote data quality (eg, double data entry; range checks for data values). Reference to where details of data management procedures can be found, if not in the protocol. **See page 13-14.**
Statistical methods	20a	Statistical methods for analysing primary and secondary outcomes. Reference to where other details of the statistical analysis plan can be found, if not in the protocol. **See page 14-15.**
	20b	Methods for any additional analyses (eg, subgroup and adjusted analyses). **See page 14-15.**
	20c	Definition of analysis population relating to protocol non-adherence (eg, as randomised analysis), and any statistical methods to handle missing data (eg, multiple imputation). **See page 14-15.**

Methods: Monitoring

Data monitoring	21a	Composition of data monitoring committee (DMC); summary of its role and reporting structure; statement of whether it is independent from the sponsor and competing interests; and reference to where further details about its charter can be found, if not in the protocol. Alternatively, an explanation of why a DMC is not needed. **See pages 18-19.**
	21b	Description of any interim analyses and stopping guidelines, including who will have access to these interim results and make the final decision to terminate the trial. **See pages 18-19.**
Harms	22	Plans for collecting, assessing, reporting, and managing solicited and spontaneously reported adverse events and other unintended effects of trial interventions or trial conduct. **See pages 18-19.**
Auditing	23	Frequency and procedures for auditing trial conduct, if any, and whether the process will be independent from investigators and the sponsor. **See pages 18-19.**

Ethics and dissemination

Research ethics approval	24	Plans for seeking research ethics committee/institutional review board (REC/IRB) approval. **See page 19.**
Protocol amendments	25	Plans for communicating important protocol modifications (eg, changes to eligibility criteria, outcomes, analyses) to relevant parties (eg, investigators, REC/IRBs, trial participants, trial registries, journals, regulators). **Can be added at Editors request?**
Consent or assent	26a	Who will obtain informed consent or assent from potential trial participants or authorised surrogates, and how (see Item 32). **See page 7.**
	26b	Additional consent provisions for collection and use of participant data and biological specimens in ancillary studies, if applicable. **N/A**
Confidentiality	27	How personal information about potential and enrolled participants will be collected, shared, and maintained in order to protect confidentiality before, during, and after the trial. **See page 18-19.**
Declaration of interests	28	Financial and other competing interests for principal investigators for the overall trial and each study site. **See page 21.**
Access to data	29	Statement of who will have access to the final trial dataset, and disclosure of contractual agreements that limit such access for investigators. **See page 21.**
Ancillary and post-trial care	30	Provisions, if any, for ancillary and post-trial care, and for compensation to those who suffer harm from trial participation. **N/A.**

For peer review only

Dissemination policy	31a	Plans for investigators and sponsor to communicate trial results to participants, healthcare professionals, the public, and other relevant groups (eg, via publication, reporting in results databases, or other data sharing arrangements), including any publication restrictions
	31b	Authorship eligibility guidelines and any intended use of professional writers. **N/A**
	31c	Plans, if any, for granting public access to the full protocol, participant-level dataset, and statistical code. **N/A**
**Appendices**		
Informed consent materials	32	Model consent form and other related documentation given to participants and authorised surrogates
Biological specimens	33	Plans for collection, laboratory evaluation, and storage of biological specimens for genetic or molecular analysis in the current trial and for future use in ancillary studies, if applicable

*It is strongly recommended that this checklist be read in conjunction with the SPIRIT 2013 Explanation & Elaboration for important clarification on the items. Amendments to the protocol should be tracked and dated. The SPIRIT checklist is copyrighted by the SPIRIT Group under the Creative Commons "Attribution-NonCommercial-NoDerivs 3.0 Unported" license.

For peer review only

BMJ Open

Supporting self-management of low back pain with an internet intervention in primary care: A protocol for a randomised controlled trial of clinical and cost-effectiveness (SupportBack 2)

Journal:	*BMJ Open*
Manuscript ID	bmjopen-2020-040543.R1
Article Type:	Protocol
Date Submitted by the Author:	04-Jun-2020
Complete List of Authors:	Geraghty, Adam; University of Southampton, Primary Care and Population Sciences<br>Roberts, Lisa; University of Southampton, Health Sciences<br>Hill, Jonathan; Keele University, Institute of primary care and health sciences<br>Foster, Nadine; Keele University, Arthritis Research UK Primary Care Centre<br>Yardley, Lucy; University of Southampton, Psychology; University of Bristol, School of Health Sciences<br>Hay, Elaine; Arthritis Research Primary Care Centre<br>Stuart, Beth; University of Southampton, Primary Care and Population Science<br>Turner, David; University of East Anglia, Public Health and Primary Care<br>Griffiths, Gareth; University of Southampton<br>Webley, Frances; University of Southampton, Southampton Clinical Trials Unit<br>Durcan, Lorraine; University of Southampton, Southampton CTU<br>Morgan, Alannah; University of Southampton, Southampton Clinical Trials Unit<br>hughes, Stephanie; University of Southampton, Primary Care and Population Sciences<br>Bathers, Sarah ; Keele University, Keele Clinical Trials Unit<br>Butler-Walley, Stephanie; Keele University, Keele Clinical Trials Unit<br>Wathall, Simon; Keele University, Institute of Primary Care & Health Sciences<br>Mansell, Gemma ; Aston University, Psychology<br>Leigh, Linda<br>Little, Paul; University of Southampton, Primary Care and Population Science;
**Primary Subject Heading**:	Rheumatology
Secondary Subject Heading:	Rehabilitation medicine
Keywords:	Back pain < ORTHOPAEDIC & TRAUMA SURGERY, PRIMARY CARE, REHABILITATION MEDICINE

For peer review only - http://bmjopen.bmj.com/site/about/guidelines.xhtml

I, the Submitting Author has the right to grant and does grant on behalf of all authors of the Work (as defined in the below author licence), an exclusive licence and/or a non-exclusive licence for contributions from authors who are: i) UK Crown employees; ii) where BMJ has agreed a CC-BY licence shall apply, and/or iii) in accordance with the terms applicable for US Federal Government officers or employees acting as part of their official duties; on a worldwide, perpetual, irrevocable, royalty-free basis to BMJ Publishing Group Ltd ("BMJ") its licensees and where the relevant Journal is co-owned by BMJ to the co-owners of the Journal, to publish the Work in this journal and any other BMJ products and to exploit all rights, as set out in our licence.

The Submitting Author accepts and understands that any supply made under these terms is made by BMJ to the Submitting Author unless you are acting as an employee on behalf of your employer or a postgraduate student of an affiliated institution which is paying any applicable article publishing charge ("APC") for Open Access articles. Where the Submitting Author wishes to make the Work available on an Open Access basis (and intends to pay the relevant APC), the terms of reuse of such Open Access shall be governed by a Creative Commons licence – details of these licences and which Creative Commons licence will apply to this Work are set out in our licence referred to above.

Other than as permitted in any relevant BMJ Author's Self Archiving Policies, I confirm this Work has not been accepted for publication elsewhere, is not being considered for publication elsewhere and does not duplicate material already published. I confirm all authors consent to publication of this Work and authorise the granting of this licence.

For peer review only

Supporting self-management of low back pain with an internet intervention in primary care: A protocol for a randomised controlled trial of clinical and cost-effectiveness (SupportBack 2)

Adam W A Geraghty[1] *, Lisa C Roberts[2], Jonathan Hill[3], Nadine E Foster[3], Lucy Yardley[4], Elaine M Hay[3], Beth Stuart[1], David A Turner[5], Gareth Griffiths[6], Frances Webley[6], Lorraine Durcan[6], Alannah Morgan[6], Stephanie Hughes[1], Sarah Bathers[7], Stephanie Butler-Walley[7], Simon Wathall[7], Gemma Mansell[8], Linda Leigh[9], Paul Little[1].

[1] Primary Care, Population Sciences and Medical Education, University of Southampton, United Kingdom.

[2] School of Health Sciences, University of Southampton, United Kingdom & University Hospital Southampton NHS Foundation Trust, United Kingdom.

[3] Primary Care Centre Versus Arthritis, School of Primary, Community and Social Care, Keele University

[4] School of Psychological Science, University of Bristol and Department of Psychology, University of Southampton, United Kingdom.

[5] Norwich Medical School, University of East Anglia, United Kingdom.

[6] Southampton Clinical Trials Unit, University of Southampton, United Kingdom.

[7] Keele Clinical Trials Unit, School of Primary, Community and Social Care, Keele University, United Kingdom.

[8] School of Life and Health Sciences, Aston University, Birmingham, United Kingdom.

[9] Patient and Public Involvement Representative, Southampton, United Kingdom.

For peer review only

*Correspondence concerning this article should be addressed to Adam W A Geraghty, Primary Care and Population Sciences, Aldermoor Health Centre, Aldermoor Close, Southampton, Hampshire, SO16 5ST. Email: A.W.Geraghty@soton.ac.uk. Tel: (+44) 2380241051

Word Count: 5289

For peer review only

For peer review only - http://bmjopen.bmj.com/site/about/guidelines.xhtml

ABSTRACT

Introduction

Self-management and remaining physically active are first-line recommendations for the care of patients with low back pain (LBP). With a life-time prevalence of up to 85%, novel approaches to support behavioural self-management are needed. Internet interventions may provide accessible support for self-management of LBP in primary care. The aim of this randomised controlled trial is to determine the clinical and cost-effectiveness of the 'SupportBack' internet intervention, with or without physiotherapist telephone support in reducing LBP-related disability in primary care patients.

Methods and analysis

A three parallel arm, multi-centre randomised controlled trial will compare three arms: 1) usual primary care for LBP; 2) usual primary care for LBP and an internet intervention; 3) usual primary care for LBP and an internet intervention with additional physiotherapist telephone support. Patients with current LBP and no indicators of serious spinal pathology are identified and invited via general practice list searches and mailouts or opportunistic recruitment following LBP consultations. Participants undergo a secondary screen for possible serious spinal pathology and are then asked to complete baseline measures online after which they are randomised to an intervention arm. Follow-ups occur at six weeks, three, six and 12 months. The primary outcome is physical function (using the Roland and Morris Disability Questionnaire] over 12-months [repeated measures design). Secondary outcomes include pain intensity, troublesome days in pain over the last month, pain self-efficacy, catastrophising, kinesophobia, health-related quality of life and cost-related measures for a full health economic analysis. A full mixed methods process evaluation will be conducted.

For peer review only - http://bmjopen.bmj.com/site/about/guidelines.xhtml

Ethics and dissemination

This trial has been approved by an NHS Research Ethics Committee (REC Ref: 18/SC/0388). Results will be disseminated through peer-reviewed journals, conferences, communication with practices and patient groups. Patient representatives will support the implementation of our full dissemination strategy.

Trial registration number: ISRCTN reference 14736486 (pre-results)

Key words: Low Back Pain; Internet Intervention; Primary Care; Randomised Controlled Trial

For peer review only

STRENGHTS AND LIMITATIONS

- The SupportBack 2 trial is a large multi-centre randomised trial that will determine the additional benefit, over usual primary care, of an internet-based approach that supports self-management of patients with LBP in UK primary care.
- The trial is designed to investigate the effectiveness of an internet intervention in addition to usual primary care, both with and without telephone physiotherapist support.
- A full mixed methods process evaluation will be carried out to inform a logic model and 'theory of change' for the interventions.
- Inclusion is limited to those with LBP who have access to the internet and are able to communicate in English without assistance.

For peer review only

INTRODUCTION

Low Back Pain (LBP) has a life-time prevalence of up to 85% [1] and is the greatest single cause of years lived with disability globally. [2] LBP is primarily managed in primary care, [3] where first-line recommendations are to self-manage and remain physically active. [4] Supporting effective behavioural self-management of LBP is increasingly important; the most recent guidelines place less emphasis on pharmacological and surgical treatments. [5] General practitioners are unlikely to have the training or the capacity to support behavioural self-management, and access to specialist musculoskeletal (MSK) services can be variable. [6] New roles such as First Contact Physiotherapists in general practice are emerging, but implementation is at an early stage. [7] Internet interventions may offer a route to rapidly scalable behavioural support for patients with LBP, however their effectiveness in UK primary care needs to be determined.

Internet interventions are typically automated, interactive, tailored interventions that make use of multimedia formats to deliver behaviour change strategies online. [8] Internet interventions are one form of a broader category of digital interventions that draw on digital technologies including the internet, mobile devices and activity sensors. [9] A recent systematic review of digital interventions for LBP highlighted substantial heterogeneity in intervention delivery, duration and outcomes, making it difficult to draw conclusions regarding effectiveness. [10] Since the publication of this review there has been a focus on mobile apps for LBP: a German study has shown that a mobile app delivering multidisciplinary self-management support for patients with LBP recruited via online advertising significantly reduced pain at 12-week follow-up, compared to a 6-week course of exercise delivered by physiotherapists plus online education. [11] An ongoing European programme of work seeks to determine the effectiveness of a mobile app-based digital decision support self-management programme (selfBACK) for patients recently consulting in primary care for LBP. [12]

For peer review only

Whilst mobile apps show potential, internet interventions likely have an accessibility advantage; they can be accessed from any device with an internet connection (e.g. desktop, laptop, tablet, mobile phone).

SupportBack is an internet intervention designed to support patients to self-manage their LBP following consultation in primary care. [13, 14] It was developed using evidence and theory in combination with the person-based approach, where systematic qualitative research is integrated throughout development. [15, 16] SupportBack is designed to be as accessible as possible, both in presentation style and in target; it can be used by people with both acute and persistent LBP. SupportBack has been developed to be used in addition to usual care, either as a stand-alone internet intervention or in combination with physiotherapist telephone support. A randomised controlled feasibility trial demonstrated both the feasibility of trial procedures and the effective delivery of the intervention and telephone support. [14] In a nested qualitative study within the feasibility trial, Geraghty et al. [15] found that patients were broadly positive about the intervention; they suggested that it provided reassurance whilst supporting becoming more physically active as a primary pain management strategy.

The aim of the present full randomised controlled trial is to determine the clinical and cost-effectiveness of the SupportBack internet intervention, delivered in addition to usual care with and without physiotherapist telephone support, in reducing LBP-related physical disability in UK primary care.

METHODS

Design

A three parallel arm (1:1:1), multicentre randomised controlled trial is being conducted to determine the clinical and cost-effectiveness of an internet intervention for patients

For peer review only

with LBP in primary care. Participants will be followed up at six weeks, three, six and 12 months.

Study setting

The trial is being carried out with patients from 140-180 general practices across the UK. Patients access the intervention through their own devices with internet access (e.g. a desktop, laptop, tablet, mobile phone) at a location that is convenient for them (e.g. at home or at work). If allocated to receive telephone physiotherapist support, this support is delivered wherever is convenient for the patient. A list of patient identification centres is available from the trial team on request.

Eligibility criteria

Inclusion criteria:

- Aged 18 and above
- Current low back pain (have experienced pain in the last week) with or without sciatica
- Access to the internet and an active email address
- Ability to read/understand English without assistance
- Ability to provide informed consent

Exclusion criteria:

- Signs and symptoms in a patient with LBP that indicate potential serious spinal pathology such as infection, malignancy, fracture, inflammatory back pain, progressive neurology and/or cauda equina.
- Have had spinal surgery in the past six months
- Pregnancy
- Taken part in the prior SupportBack feasibility study

For peer review only

Identification, recruitment and screening

Two recruiting centres, Southampton and Keele [each with a team of telephone support physiotherapists] are working with National Institute for Health Research (NIHR) Clinical Research Networks (CRNs) to facilitate the recruitment of general practices. Potentially eligible participants will be identified in one of two ways:

1) Patients who have consulted with LBP in the last two months will be identified by general practice staff from computerised records of consultations. Practices will be asked to repeat the searches approximately three times, or until the target number of patients per practice has been reached. Resulting lists of patients identified by the search will be screened by a practice GP who will rule out patients based on aspects of the eligibility criteria that can be determined from patient notes.

2) During a patient consultation and on entering a relevant diagnostic or symptom Read code into the patient electronic medical record, GPs will be prompted about the trial and patient eligibility by an automated 'pop-up' screen activated by the Read code. GPs will then screen for eligibility [using the inclusion/exclusion criteria listed] and patients identified as suitable will have their medical record electronically tagged. A download of 'tagged' patients will occur regularly, anticipated to be every two weeks. This method will be used in practices where possible. Participating general practices not implementing the 'pop up' Read code method can identify potential patients during consultation. Having considered eligibility the GP or Nurse Practitioner will provide the patient with an invitation pack.

Patients identified either by a medical records review or general practice consultation are mailed a study pack including an invitation letter from the GP, participant information sheet, reply slip, screening questions and pre-paid envelope. Interested

For peer review only - http://bmjopen.bmj.com/site/about/guidelines.xhtml

patients return the reply slip and screening questions using the pre-paid envelope to the Research Team. Screening consists of two questions regarding current LBP and access to the internet, followed by three safety questions listing symptoms that may indicate serious spinal pathology. Patients who answer 'Yes' to the first two questions, and 'No' to all safety questions, are considered eligible. For those who complete the screening questions and fail safety screening, a physiotherapist contacts the patient to make an appropriate clinical recommendation on hearing a further description of the symptoms. Those who fail the screening are documented on a screening log maintained by the Research Team. All patients considered eligible for the trial are assigned a unique participant identification number and sent a link to the study website, to complete consent, baseline questionnaires and be randomised. Recruitment opened in November 2018 and is expected to close in December 2020, with data collection completing approximately 12 months later in December 2021.

Randomisation, allocation and blinding

The randomisation process for this trial is fully automated. The intervention and data collection software automatically generates the randomisation sequence, and a computer-generated algorithm block randomises participants to the trial groups. Participants are being stratified by trial recruiting centre and level of physical function: a score of less than four on the Roland Morris Disability Questionnaire (RMDQ [17]) is being used to denote a lower level of self-rated physical disability. As the automated software randomises patients, the randomisation sequence is concealed from the trial team. Patients are automatically informed of their group allocation through the internet intervention software. As patients are engaging with a behavioural intervention, they are not blind to allocation. The majority of data will be collected online, or by post. Telephone calls are used to collect primary outcome data where there has been no response to online and postal follow-up. The callers are blind to group allocation. The statisticians conducting the analyses will remain blind to group allocation. The health economist will conduct the majority of analysis blinded to group, however, estimates

For peer review only - http://bmjopen.bmj.com/site/about/guidelines.xhtml

of total cost require the addition of costs specific to the provision of the interventions so will become un-blinded at this point. Figure 1 details patient flow through the trial.

[Insert Figure 1 about here]

Interventions

Usual care

Participants randomised to this arm will continue to receive unrestricted usual primary care for LBP. Current NICE recommendations for primary care management of LBP suggest assessment to rule out specific spinal pathology and use of risk stratification tools (e.g. STarT Back [18]). Guidelines with regard to pharmacotherapy recommend NSAIDs at the lowest effective doses for the shortest period of time. Paracetamol and opiates are not recommended for routine use. Care may also include referrals for physiotherapy and psychological interventions such as cognitive behaviour therapy (CBT). Despite guidelines, there is likely to be a variety in what is provided for patients as part of usual care over the trial period from no further contacts, to referrals to physiotherapy and pain clinics. Consequently, health care resource use will be documented and form a central part of our health economic analysis.

Usual care + Internet intervention

Participants randomised to this arm will continue to receive unrestricted usual primary care. In addition, they will have access SupportBack. SupportBack is a multi-session, interactive internet intervention that provides participants with accessible information, advice and tools to support the effective self-management of LBP. The SupportBack intervention [including telephone support] has been extensively described elsewhere. [13, 15] In brief, the central components of the intervention include graded goal setting, self-monitoring, and tailored feedback to encourage physical activity/exercise increases or maintenance. SupportBack also provides educational modules regarding pain and LBP-related topics (relieving pain; flare ups; work; sleep; mood; daily living).

For peer review only

Throughout the digital material, there is a focus on supporting motivation for behaviour change: Techniques employed include reassuring about consequences of movement, modelling of use of activity as a primary pain management strategy and using automation to deliver positive feedback through reassurance regarding consequences. These techniques were combined with a person-based approach in the development of SupportBack, where the application of such approaches was guided by systematic in-depth qualitative research with patients with LBP. [16]

Practically, patients can access SupportBack from any device with an internet connection from wherever is most convenient for them. SupportBack consists of six sessions, and patients are encouraged to log in and use one session per week. Automated reminders adhere to this schedule. The first session highlights the centrality of physical activity in managing LBP, and supports patients to set goals to either walk more, or engage with a range of gentle back exercises of their choice. Goal options are tailored and are based the extent that patients report their LBP obstructs their day-to-day activities. The further sessions feature self-monitoring and feedback regarding their progress with walking or exercise goals, combined with encouragement from SupportBack to continue. After the first session, patients can unlock one further module per week on topics such as sleep, mood and work. These build into a personal repository, that alongside weekly goals, can be accessed at any time. If engaged with weekly, the tailored, interactive part of the intervention will last six weeks. Following completion of all the sessions, SupportBack converts into static resource where all activities/exercises and modules can be accessed for the duration of the trial.

Usual care + internet intervention + telephone physiotherapist support

Participants randomised to this arm will also continue to receive unrestricted usual primary care, with access to the SupportBack internet intervention. In addition, these participants will also receive up to one hour of physiotherapist support over the telephone (the first call can be up to 30 minutes, with two follow-up calls of up to 15

For peer review only

minutes, over the six-week intervention period). At both centres (Southampton and Keele) support is provided by musculoskeletal physiotherapists working in the NHS.

The objectives of the telephone contact are to encourage the use of the SupportBack intervention, provide reassurance regarding LBP and encourage adherence to physical activity goals. The physiotherapists are asked to closely adhere to a standardised content checklist for each phone call. The checklist follows the CARE approach [19] (Congratulate, Ask, Reassure, Encourage), developed specifically to guide support for digital interventions. Drawing on existing clinical skills, it ensures a general supportive approach and requires minimal training (all support physiotherapists attended a two-hour training session ahead of the trial). Whilst telephone physiotherapists are able to address individual participant concerns, they are asked to avoid additional individualised participant assessment and treatment recommendations beyond the internet intervention content. Physiotherapists complete a checklist for each call. Checklists are returned to the trial coordinating centre, where intervention fidelity will be assessed.

Measures

All measures and time points for collection are listed in Table 1.

Primary outcome

The primary outcome in this trial is LBP-related physical function measured with the Roland Morris Disability Questionnaire. [17] Function forms a central domain in the recommended core outcomes set for LBP trials. [20] The RMDQ is a recommended measure of physical function [21] and is commonly used in primary care LBP trials.

Secondary measures

For peer review only

Demographic data is being collected at baseline including age, sex, educational attainment, marital and occupational status. A range of secondary measures are being collected including pain intensity, [22] number of troublesome days in pain [23] and risk of pain related disability, [24] Pain-related psychological variables are being measured including kinesiophobia [fear of movement], [25] catastrophizing, [26] pain self-efficacy, [27] outcome expectations [28] and symptoms of depression and anxiety. [29] General physical activity is being measured with the Godin Leisure-time Exercise Questionnaire [30] in this trial; the short-form IPAQ [31] was used in the feasibility trial but produced unreliable data. We are also measuring intervention specific physical activity with a single item developed for this trial. Adherence to walking and physical activity goals are being measured with specifically developed items, based closely on measures previously used in a related behavioural trial [32] and difficulties with the intervention are being measured using the Problematic Experiences of Therapy Scale. [33]

To support the health economic analysis health-related quality of life is being measured with the EQ-5D-5L. [34] All resources required to provide the internet intervention and the telephone support will be recorded. Details of NHS resource use will be recorded from general practice notes review. This will include both primary and secondary care contacts and will cover both general health care usage in addition to LBP specific care in the follow-up period. Additionally, LBP specific medication use will be captured. There may also be differences in LBP related services paid for by study participants: for example, complementary or alternative medicine (CAM). Participants may also require time off work. Additionally, there may be under-reporting of LBP-specific resource use from medical records. These resources will be captured by means of a simple questionnaire administered at six and 12 months. The time-off work question and items relating to use of private health care will additionally be asked at baseline. All resources identified will be costed using appropriate local and national data, for example NHS reference costs and Unit Costs of Health and Social Care. Occupational

For peer review only

status is being measured with a brief questionnaire developed for this trial.

The internet intervention software automatically collects data on number of logins, page and module views and time spent in each login. This data will be used to explore adherence and user engagement to the digital component of the intervention.

Table 1. Outcomes and measures used in the trial

Domain	Measure	Time point
Function (Primary outcome)		
LBP-related physical function	Roland Morris Disability Questionnaire (RMDQ) [17]	Baseline, 6 weeks, 3, 6, 12 months. All arms.
Pain		
Pain intensity	Pain index (Numerical rating scales measuring current, average and least pain over the last 2 weeks) [22]	Baseline, 6 weeks, 3, 6, 12 months. All arms.
Pain duration	Time since last pain free month [35]	Baseline. All arms.
Troublesomeness of pain	Troublesome days in pain over the last month [developed from days in pain measure [23]	Baseline, 6 weeks, 3, 6, 12, months. All arms.
Risk of persistent pain-related disability	STarT Back tool [24]	Baseline, 12 months. All arms.
Psychological processes related to pain		
Fear of movement	Tampa Scale for Kinesiophobia (TSK-11) [25]	Baseline, 12 months. All arms.
Catastrophising/negative orientation towards pain	Pain Catastrophizing Scale (PCS) [26]	Baseline, 12 months follow-up All arms.
Confidence in ability to manage pain	Pain Self-Efficacy Questionnaire (PSEQ) [27]	Baseline, 6 weeks, 3, 6, 12 months. All arms.
Self-efficacy for managing LBP	Single item from Musculoskeletal Health Questionnaire (MSK-HQ) [36]	Baseline, 6 weeks, 3, 6, 12 months. All arms.

For peer review only

Outcome expectation	Expectancy question from Credibility and Expectancy Questionnaire modified for LBP CEQ low back pain modification [28, 37]	Baseline, all arms. following session one of SupportBack Internet intervention arms only.
Mental health	Patient Health Questionnaire-4 [29] depression and anxiety measure	Baseline, 12 months. All arms.
Physical activity/adherence		
General physical activity	Godin leisure-time exercise questionnaire [30]	Baseline, 12 months. All arms
SupportBack-related physical activity	Single item measure developed for the trial	Baseline, 6 weeks, 3, 6, 12 months. All arms.
Adherence to back-specific activity	Item developed for this trial, based on previous behavourial adherence measures [32]	12 months. All arms
Difficulties with intervention recommendations	Problematic Experiences of Therapy Scale (PETS) [33]	12 months.<br>Internet intervention arms only.
Satisfaction and enablement		
Satisfaction with care received for LBP	Single satisfaction item developed for trial	6 weeks. All arms.
Enablement	Patient Enablement Instrument (PEI) [38]	6 weeks, 12 months. All arms.
Health related quality of life, health care resource use, and occupational status		
Health-related Quality of Life	ED-5D Five Level [34]	Baseline, 6 weeks, 3, 6, 12 months. All arms.
Use of over-the-counter (OTC) medication for LBP	Single item measuring self-reported OTC medication usage for LBP	Baseline, 6 months, 12 months. All arms.
Participant borne costs	Participant reported health resource use questionnaire developed for this study	Baseline, 6 months, 12 months. All arms.
NHS health care resource use (specific to back pain, and general)	General practice medical notes review and Participant reported health care resource use questionnaire developed for this trial	Baseline, 6 months, 12 months. All arms.
Occupational impact of LBP	Brief occupational questionnaire developed for this trial.	12 months.<br>All arms.
Use of internet resources		

For peer review only - http://bmjopen.bmj.com/site/about/guidelines.xhtml

Use of internet resources	Single item regarding use of internet resources for LBP over trial period.	12 months.<br>All arms.

Sample size

The reported minimally clinical important difference (MCID) between groups for the RMDQ varies. A between group MCID of 2 or 3 points is commonly reported. [23, 39, 40] However, it has been suggested that a difference of 1.5 may still be important, particularly in the context of low intensity interventions. [23] Whether delivered as a stand-alone intervention or coupled with brief telephone support, SupportBack is a low intensity intervention with the potential to be rapidly scalable. Consequently, we considered a between group change of at least 1.5 to be a meaningful difference in this context. For our repeated measures primary outcome, a difference of 1.5 points on the RMDQ over the follow-up period of 12 months, assuming a standard deviation of 5 in line with the feasibility study, [23] gives an effect size of 0.30. Alpha will be set to 0.025 to allow both interventions to be independently compared with the usual care alone arm. Using four repeated measures (six weeks, three, six and 12 months), and assuming a correlation between repeated measures of 0.7 and 90% power, requires 215 participants per arm. Allowing for 20% loss to follow up, this gives a total sample size of 806.

Data collection and management

Data are primarily being collected online. The LifeGuide intervention and data system collects consent, baseline data including demographics and follow-up data across the four time points (six weeks, three, six and 12 months). When first sent a link to the system following screening, if patients do not log on within a week, they are emailed to check that they received the link and advised to look in their spam mail. If there is no response, one telephone call is attempted by the research team.

For peer review only

With regard to follow-up protocol, where there is no response to the online follow-up questionnaire emails, two reminder emails and text messages will be sent. Following continued non-response, a paper questionnaire pack with a pre-paid envelope will be sent one week after the last email/text reminder. If the paper questionnaires are not returned within two weeks of being sent, a blinded research assistant will call the participant to complete the primary outcome measure (RMDQ), quality of life questionnaire (EQ-5D-5L) and pain severity. If the participant is happy to continue, further measures from the questionnaire battery at the respective follow-up point will be collected in this manner. The full follow-up protocol with the telephone calls will be implemented at six week and 12 month follow-up points. These two follow-up points are considered most important, capturing initial and long-term response. Calling at all time points may lead to increased dropout at later time points. Follow-up at three and six months will include all the above steps except for the phone calls. All participants will receive a £5 voucher when asked to complete questionnaires at the more distant time-points of six and 12 months. Examples of data collection forms can be provided by the trial team on request.

Statistical methods

Quantitative analysis will begin following cleaning and inspection of the data. Descriptive analysis will be conducted to determine outliers and distributions of the data. Where necessary, if data are not normally distributed, transformations will be applied or another appropriate distribution used. The primary analysis for the RMDQ score will be performed using a multilevel mixed model (MLMM) framework with observations at six weeks, three, six and 12 months (level one) nested within participants (level two). Results will be reported adjusting for baseline severity in function, stratification factors and any pre-specified confounders. The model will use all the observed data and makes the assumption that missing RMDQ scores are missing at random given the observed data.

For peer review only

As there may not be a constant treatment effect over time, a treatment/time interaction will be modelled and included if significant (at the 5% level), with time treated as a random effect. An unstructured covariance matrix will be used.

Analysis of secondary outcomes will also be conducted using linear regression for continuous outcomes and logistic regression for dichotomous outcomes, again controlling for baseline symptom severity, stratification factors and any potential confounders. The structure and pattern of missing data will be examined, if appropriate, and a sensitivity analysis based on data imputed using a multiple imputation model presented. Data will be analysed on an intention-to-treat basis [they will be analysed as randomised]. We will also undertake a complier-average causal effect (CACE) analysis, [41] which compares compliant participants in the intervention group, with those in the control group whose characteristics are similar enough to the intervention group compliers to suggest they too would have complied with the intervention, given the opportunity to do so. Compliance for these analyses in the intervention arm will be defined as completing at least Session 1 of the internet intervention. Session 1 contains the central rationale for the intervention; that physical activity is primary in the management of LBP and provides instructions and advice on goal setting. The latter sessions follow a similar format to the first introductory module. With regard to the physiotherapist telephone support arm, we consider per protocol to be receiving at least two of the three planned phone calls. The telephone element is designed to be pragmatic with the necessary flexibility to fit patients' requirements. However, receiving at least two of three calls indicates that support was delivered over time; an important aspect in the design and integration with the internet intervention.

It is not anticipated that there will be significant practice level [cluster] effects but this assumption will be tested by comparing a fixed effect model to a random effects model. If there are significant practice level effects then, the model will include a random effect for practice [random intercept] and participant [random intercept and slope on time] to

For peer review only

allow for between participant and practice differences at baseline and between participant differences in the rate of change over time (if significant at the 5% level), and fixed effects for baseline covariates.

No interim analyses are planned. Full details of the analyses to be undertaken will be set out in the Statistical Analysis Plan and approved by the Trial Steering Committee (TSC). Our full statistical analysis plan will be published on the trial website in due course (https://www.southampton.ac.uk/medicine/academic_units/projects/supportback2.page).

Cost-effectiveness analysis

A 'within trial' economic analysis will be conducted alongside the RCT to estimate the incremental cost-effectiveness of the SupportBack 2 interventions compared to usual care. The base case perspective will be that of the NHS, but other resources relevant to LBP will be collected to enable additional analysis from a societal perspective.

The main outcome measure in the economic evaluation will be the quality adjusted life year (QALY), obtained from the EQ-5D-5L instrument using the published UK value set. In addition, a cost-effectiveness analysis will be carried out using the study primary outcome measure, i.e. the cost per point change in back-related physical function measured using the RMDQ will be estimated. Both costs and effects will be estimated using multiple regression, to allow for potential confounders, such as baseline scores for EQ-5D-5L and RMDQ. Standard practice will be followed to calculate incremental cost-effectiveness ratios (ICERs), and present ICER(s) where any one option has both higher costs and increased effects compared to another. ICERs will show incremental cost per QALY or incremental cost per point improvement in RMDQ. Bootstrapping will be used to calculate cost-effectiveness acceptability curves (CEACs). These will illustrate the effect of uncertainty on study results. Major

For peer review only

assumptions made in the analysis will be tested by means of sensitivity analysis. In particular, assumptions made during the costing of the intervention such as the number of individuals who will be using the website will be explored. Similar methods to the main clinical analysis will be used to handle missing data, i.e., analysis of patterns of missing data with multiple imputation methods employed if deemed appropriate. The proposed health economics analysis will be detailed in a health economics analysis plan (HEAP) which will be completed before analysis commences. The HEAP will be circulated for comment prior to the health economics analysis. Any digressions from the HEAP will be documented and justified in the final health economics report.

Process evaluation

A process evaluation will be carried out following Medical Research Council (MRC) guidelines on process evaluations of complex interventions. [42] In order to provide a detailed understanding of the SupportBack intervention three aspects will be examined: Implementation, mechanisms of impact (mediators) and context (moderators). A mixed methods approach will be used to explore these elements.

Implementation

Quantitative data describing trial implementation will be presented including number of practices recruited, patient eligibility [including reasons for declined participation where possible, and analysis of screen failures] and recruitment rates. The number of withdrawals from the trial per arm will be presented, along with numbers/percentages of dropouts from the intervention who do not respond to follow-up. Use of the internet intervention will be described by presenting automated data collected on number of logins and modules accessed for both the internet intervention and the intervention plus telephone physiotherapist support arm. With regard to the internet intervention plus telephone physiotherapist support arm, the number of support calls successfully made (and attempts contact the patient), along with the mean number per

For peer review only

participant in this arm will be described.

Qualitative interviews will be conducted with up to 45 trial participants following the three, six and 12-month follow-up points. Different participants will be interviewed at each time point, enabling us to explore how time since accessing the tailored weekly component of the intervention effects how suggestions are used and implemented in daily life. Interviews will also be conducted with the trial physiotherapists. Participants will be purposively sampled to ensure diversity in terms of age, sex and symptom severity (physical function, pain intensity and duration). Participants will also be sampled based on high and low usage of the internet intervention and high and low engagement with the telephone physiotherapist support. For participants, questions will focus on their experience of using the intervention, including telephone physiotherapist support and usual care. Interviews with the trial support physiotherapists will be designed to explore their experience of delivering the intervention, with a particular focus on barriers and facilitators, and determinants of successful exchanges.

Mechanisms of impact

A logic model of proposed mechanisms affecting LBP-related physical disability and pain outcomes for the SupportBack intervention has been developed (see [14]). This model will be used as the basis of both quantitative and qualitative exploration of mechanisms. Quantitative analyses will focus on psychological and behavioural mechanisms influencing outcome following use of the interventions including expectancy, self-efficacy to manage LBP, physical activity, self-reported goal setting across the intervention and objective measures of intervention use (sessions completed, use of additional modules, e.g. mood, sleep etc.). In order to explore whether two core mechanisms' [mediating variables] contribution to outcome is unique to the internet intervention arms, brief single items capturing self-efficacy (SE) and physical activity (PA) are being measured in all three arms (including usual care). SE and PA are being

For peer review only - http://bmjopen.bmj.com/site/about/guidelines.xhtml

measured at baseline and in the outcome questionnaire sets at six weeks, three, six, and 12 months. Mediation analysis will be used to explore relationships between mediating variables and LBP-related physical function and pain intensity across the 12-month follow-up period. We will also explore the potential of multilevel modelling to examine mediating variables association with the outcome over time. [43] Appropriate checks of the assumptions of causal modelling, such as exchangeability (no confounding), consistency, effect modification and temporality will also be carried out. [44, 45]

Questions will be included in the qualitative interviews focusing on participants' perceptions of how use of the SupportBack intervention and/or telephone support affected their LBP. This will enable the inductive exploration of participants' views and triangulation of qualitatively derived theory on mechanism with our quantitative analysis. Similar questions will also be explored in the usual care arm, focusing on how elements of their usual care may have led to improvements in their LBP.

Context

The relationship between elements of participants' context [moderators] and the effect of the interventions across the 12-month follow-up period will be explored. This will include variables such as LBP severity and duration at baseline, age, educational level and occupation status. Following the analysis of mechanisms, correlations and multiple regression (linear and logistic) will be used to explore relationships between moderating variables and LBP-related physical function and pain intensity. Qualitatively, the above aspects of participants' context, including their own descriptions of their LBP history, will feed into analysis when exploring themes regarding participants use of the intervention and their perceptions of benefit. [46]

Qualitative analysis

For peer review only - http://bmjopen.bmj.com/site/about/guidelines.xhtml

Interview data collected regarding implementation, mechanisms and context will be transcribed verbatim, coded and analysed using an inductive thematic analytic approach. [47, 48] This will ensure participants' qualitative data are not constrained by the direction of a particular theoretical model, and enable novel insights from qualitative work to be added into the theory-driven logic model. A key aspect of the qualitative analysis will entail exploring differences in accounts at different time points. This will enable us explore how time since the tailored weekly sessions impacts on the process of self-management.

Data monitoring and confidentiality

The SupportBack 2 trial has a data monitoring and ethics committee (DMEC) composed of a statistician (chair) and two academic clinicians (Professor in Primary Care Research and Professor of Physiotherapy respectively). The DMEC reports to the Trial Steering Committee (TSC) and is fully independent from the trial Sponsor with no competing interests. Interim descriptive analyses are prepared for the DMEC. The DMEC charter can be obtained from the research team on request.

All Serious Adverse Events (SAEs) are reported to the lead Clinical Trial Unit (CTU). The assessment of seriousness will be made by the participants GP or delegate. Assessment of causality will be made by the GP or delegate, and related or unrelated status will be determined. As the SupportBack intervention provides reassurance and encourages gentle activity within the participants' own limits, there are no 'expected' SAEs documented.

All patient data are being kept in strict confidence and managed in accordance with the Data Protection Act 2018 and General Data Protection Regulation (GDPR 2018) legislation. The University of Southampton policy on archiving will be followed; the data will be stored for 10 years following the end of the study, after which time it will

For peer review only - http://bmjopen.bmj.com/site/about/guidelines.xhtml

be disposed of securely. Following completion of the trial, a cleaned anonymised data set will be shared on request.

Patient and public involvement (PPI)

Patient representatives have been involved with the SupportBack trials from the outset. The idea for the trials and their subsequent design was informed by the local branch of the national charity BackCare. From this group, LL joined the research team and contributed to funding applications for both feasibility and main trials. SupportBack 2 has a panel of three PPI representatives who are part of the trial management group (TMG), advising on patient facing materials and contributing to discussions of trial related issues as they arise. PPI will pay a key role in dissemination of trial findings and interpretation of qualitative data.

Ethics and dissemination

The SupportBack 2 trial has received full ethical approval form a local review board (REC Ref: 18/SC/0388). All potentially eligible patients receive a patient information sheet. This information emphasises that participation in the trial is voluntary and that the participant may withdraw from the trial at any time for any reason. The participants are given the opportunity to ask any questions that may arise by speaking with the trial team and time to consider the information fully prior to agreeing to participate.

The findings of this trial will be published in peer reviewed journals and presented at international conferences. We will develop press releases in order to disseminate the findings to the general public, and work closely with our PPI collaborators to ensure dissemination to patient and other special interest groups. A summary of the findings will be sent to all included general practices and those patients that request this information. If the intervention is shown to be effective, we will work with developers to rapidly develop a version for widescale dissemination and implementation.

For peer review only

Author contributions

AWAG, PL, LCR, NEF, JCH, LY, DAT, EMH and GG designed the trial and secured funding. AWAG led the development of the internet intervention with LY and LCR, with further input from NEF, JCH, EMH, PL and LL. LY, AWAG, LCR, JCH, NEF, developed the physiotherapy telephone support package. FW, LD, AM, SH, SB, SBW and SW oversee, manage and coordinate all aspects of the trial and data acquisition. BS wrote the statistical analysis plan reported in the protocol with input from GM, and DAT wrote the health economic plan. LL is a public contributor who as contributed substantially to all aspects of this project since inception. AWAG drafted the manuscript with revision and input from PL, LCR, NEF, JCH, LY, DAT, EMH, GG, FW, LD, AM, SH, SB, SBW, SW, BS, LL & GM. AWAG is the guarantor.

Acknowledgements

There are a number of individuals we would like to thank for their ongoing support for this trial: Alastair Mobley at the CRN West Midlands for help with recruitment; Anna Thornhill, Emma Mcloughlin, Jacqui Tomkins, Zoe Oram, Caroline Harwood, for screening patients; Jessica White, Julie Thorpe, Kathryn Palmer, Sarah Olivant, Michael Gillingham, Katrina Humphreys, Lucy Huckfield, Tina Hadley-Barrows, and Yvonne Rimmer for providing telephone support; Dr Sarah Williams and Solent NHS Trust for supporting key setup and trial processes. SupportBack was developed using LifeGuide software and methodologies, which was partly funded by the NIHR Southampton Biomedical Research Centre (BRC).

Funding statement

For peer review only

This work was supported by the National Institute of Health Research (NIHR) Health Technology Assessment (HTA) programme, grant number 16/111/78. This protocol describes independent research funded by the NIHR. The views expressed are those of the author[s] and not necessarily those of the NHS, the NIHR or the Department of Health and Social Care. NEF was funded by an NIHR Research Professorship (NIHR-RP-011-015), LCR was funded by a NIHR Senior Clinical Lectureship (round 3) and NEF, EMH and PL are NIHR Senior Investigators. LY is an NIHR Senior Investigator and her research programme is partly supported by NIHR Applied Research Collaboration (ARC)-West, NIHR Health Protection Research Unit (HPRU) for Behavioural Science and Evaluation, and the NIHR Southampton Biomedical Research Centre (BRC)

Competing interests statement

Authors declare no competing interests.

Access to data

Data will be available to access from the authors on reasonable request.

REFERENCES

1. Andersson GB. Epidemiological features of chronic low-back pain. Lancet. 1999;354:581-585.
2. Hoy D, March L, Brooks P et al. The global burden of low back pain: estimates from the Global Burden of Disease 2010 study. Ann Rheum Dis. 2014;73:968-974.

For peer review only - http://bmjopen.bmj.com/site/about/guidelines.xhtml

3. Koes BW, van Tulder M, Lin CW, Macedo LG, McAuley J, Maher C. An updated overview of clinical guidelines for the management of non-specific low back pain in primary care. Eur Spine J. 2010;19:2075-2094.

4. National Institute for Health and Care Excellence. Low back pain and sciatica in over 16s: assessment and management. London: NICE; 2016

5. Foster NE, Anema JR, Cherkin D et al. Prevention and treatment of low back pain: evidence, challenges, and promising directions. Lancet. 2018;391:2368-2383.

6. Salisbury C, Foster NE, Hopper C et al. A pragmatic randomised controlled trial of the effectiveness and cost-effectiveness of 'PhysioDirect' telephone assessment and advice services for physiotherapy. Health Technol Assess. 2013;17:1-157, v.

7. Improvement NHSEANHS. Elective care high impact interventions: First contact practitioner for MSK services. 20191-20.

8. Ritterband LM, Gonder-Frederick LA, Cox DJ, Clifton AD, West RW, Borowitz SM. Internet interventions: In review, in use, and into the future. Prof Psychol Res Pr. 2003;34:527.

9. Yardley L, Spring BJ, Riper H et al. Understanding and promoting effective engagement with digital behavior change interventions. Am J Prev Med. 2016;51:833-842.

10. Nicholl BI, Sandal LF, Stochkendahl MJ et al. Digital Support Interventions for the Self-Management of Low Back Pain: A Systematic Review. J Med Internet Res. 2017;19:e179.

11. Toelle TR, Utpadel-Fischler DA, Haas KK, Priebe JA. App-based multidisciplinary back pain treatment versus combined physiotherapy plus online education: a randomized controlled trial. NPJ Digit Med. 2019;2:34.

12. Sandal LF, Stochkendahl MJ, Svendsen MJ et al. An App-Delivered Self-Management Program for People With Low Back Pain: Protocol for the selfBACK Randomized Controlled Trial. JMIR Res Protoc. 2019;8:e14720.

For peer review only

13. Geraghty AWA, Stanford R, Little P et al. Using an internet intervention to support self-management of low back pain in primary care: protocol for a randomised controlled feasibility trial (SupportBack). BMJ Open. 2015;5:e009524.

14. Geraghty AWA, Stanford S, Stuart B et al. Using an internet intervention to support self-management of low back pain in primary care: Findings from a randomised controlled feasibility trial (SupportBack). BMJ Open. 2018;8:e016768.

15. Geraghty AWA, Roberts LC, Stanford R et al. Exploring Patients' Experiences of Internet-Based Self-Management Support for Low Back Pain in Primary Care. Pain Med. 2019

16. Yardley L, Morrison L, Bradbury K, Muller I. The person-based approach to intervention development: application to digital health-related behavior change interventions. J Med Internet Res. 2015;17:e30.

17. Roland M, Fairbank J. The Roland-Morris Disability Questionnaire and the Oswestry Disability Questionnaire. Spine [Phila Pa 1976]. 2000;25:3115-3124.

18. Hill JC, Dunn KM, Main CJ, Hay EM. Subgrouping low back pain: a comparison of the STarT Back Tool with the Orebro Musculoskeletal Pain Screening Questionnaire. Eur J Pain. 2010;14:83-89.

19. Bradbury K, Morton K, Band R et al. Understanding how primary care practitioners perceive an online intervention for the management of hypertension. BMC Med Inform Decis Mak. 2017;17:5.

20. Chiarotto A, Deyo RA, Terwee CB et al. Core outcome domains for clinical trials in non-specific low back pain. Eur Spine J. 2015;24:1127-1142.

21. Chiarotto A, Boers M, Deyo RA et al. Core outcome measurement instruments for clinical trials in nonspecific low back pain. Pain. 2018;159:481-495.

22. Hill JC, Dunn KM, Lewis M et al. A primary care back pain screening tool: identifying patient subgroups for initial treatment. Arthritis Rheum. 2008;59:632-641.

For peer review only

23. Little P, Lewith G, Webley F et al. Randomised controlled trial of Alexander technique lessons, exercise, and massage [ATEAM] for chronic and recurrent back pain. BMJ. 2008;337:a884.
24. Hill JC, Whitehurst DG, Lewis M et al. Comparison of stratified primary care management for low back pain with current best practice [STarT Back]: a randomised controlled trial. Lancet. 2011;378:1560-1571.
25. Tkachuk GA, Harris CA. Psychometric properties of the Tampa Scale for Kinesiophobia-11 [TSK-11]. J Pain. 2012;13:970-977.
26. Picavet HS, Vlaeyen JW, Schouten JS. Pain catastrophizing and kinesiophobia: predictors of chronic low back pain. Am J Epidemiol. 2002;156:1028-1034.
27. Nicholas MK. The pain self-efficacy questionnaire: Taking pain into account. Eur J Pain. 2007;11:153-163.
28. Devilly GJ, Borkovec TD. Psychometric properties of the credibility/expectancy questionnaire. J Behav Ther Exp Psychiatry. 2000;31:73-86.
29. Kroenke K, Spitzer RL, Williams JB, Löwe B. An ultra-brief screening scale for anxiety and depression: the PHQ-4. Psychosomatics. 2009;50:613-621.
30. Godin G, Shephard RJ. A simple method to assess exercise behavior in the community. Can J Appl Sport Sci. 1985;10:141-146.
31. Craig CL, Marshall AL, Sjostrom M et al. International physical activity questionnaire: 12-country reliability and validity. Med Sci Sports Exerc. 2003;35:1381-1395.
32. Yardley L, Kirby S. Evaluation of booklet-based self-management of symptoms in Meniere disease: a randomized controlled trial. Psychosom Med. 2006;68:762-769.
33. Kirby S, Donovan-Hall M, Yardley L. Measuring barriers to adherence: validation of the problematic experiences of therapy scale. Disabil Rehabil. 2014
34. Oppe M, Devlin NJ, van Hout B, Krabbe PF, de Charro F. A program of methodological research to arrive at the new international EQ-5D-5L valuation protocol. Value Health. 2014;17:445-453.

For peer review only

35. Dunn KM, Croft PR. The importance of symptom duration in determining prognosis. Pain. 2006;121:126-132.

36. Hill JC, Kang S, Benedetto E et al. Development and initial cohort validation of the Arthritis Research UK Musculoskeletal Health Questionnaire [MSK-HQ] for use across musculoskeletal care pathways. BMJ Open. 2016;6:e012331.

37. Smeets RJ, Beelen S, Goossens ME, Schouten EG, Knottnerus JA, Vlaeyen JW. Treatment expectancy and credibility are associated with the outcome of both physical and cognitive-behavioral treatment in chronic low back pain. Clin J Pain. 2008;24:305-315.

38. Howie JG, Heaney DJ, Maxwell M, Walker JJ. A comparison of a Patient Enablement Instrument [PEI] against two established satisfaction scales as an outcome measure of primary care consultations. Fam Pract. 1998;15:165-171.

39. Bombardier C, Hayden J, Beaton DE. Minimal clinically important difference. Low back pain: outcome measures. J Rheumatol. 2001;28:431-438.

40. UK BEAMTT. United Kingdom back pain exercise and manipulation [UK BEAM] randomised trial: effectiveness of physical treatments for back pain in primary care. BMJ. 2004;329:1377.

41. Knox CR, Lall R, Hansen Z, Lamb SE. Treatment compliance and effectiveness of a cognitive behavioural intervention for low back pain: a complier average causal effect approach to the BeST data set. BMC Musculoskelet Disord. 2014;15:17.

42. Moore GF, Audrey S, Barker M et al. Process evaluation of complex interventions: Medical Research Council guidance. BMJ. 2015;350:h1258.

43. Mansell G, Hill JC, Main CJ, Von Korff M, Van Der Windt D. Mediators of treatment effect in the back in action trial: using latent growth modeling to take change over time into account. Clin J Pain. 2017;33:811.

44. Lee H, Herbert RD, Lamb SE, Moseley AM, McAuley JH. Investigating causal mechanisms in randomised controlled trials. Trials. 2019;20:524.

45. How can we design low back pain intervention studies to better explain the effects of treatment [editorial]. Spine [Phila Pa 1976] 2014;39[5]:E305.

For peer review only

46. Rogers PJ. Using programme theory to evaluate complicated and complex aspects of interventions. Evaluation. 2008;14:29-48.

47. Braun V, Clarke V. Using thematic analysis in psychology. Qual Res Psych. 2006;3:77-101.

48. Joffe H, Yardley L. Content and thematic analysis. In: Marks DF, Yardley L, editors. Research Methods for Clinical and Health Psychology. London: Sage; 2004. p. 56-68.

For peer review only

FIGURE LEGEND

Figure 1. Flow through the trial.

For peer review only

Figure 1. Flow through the trial

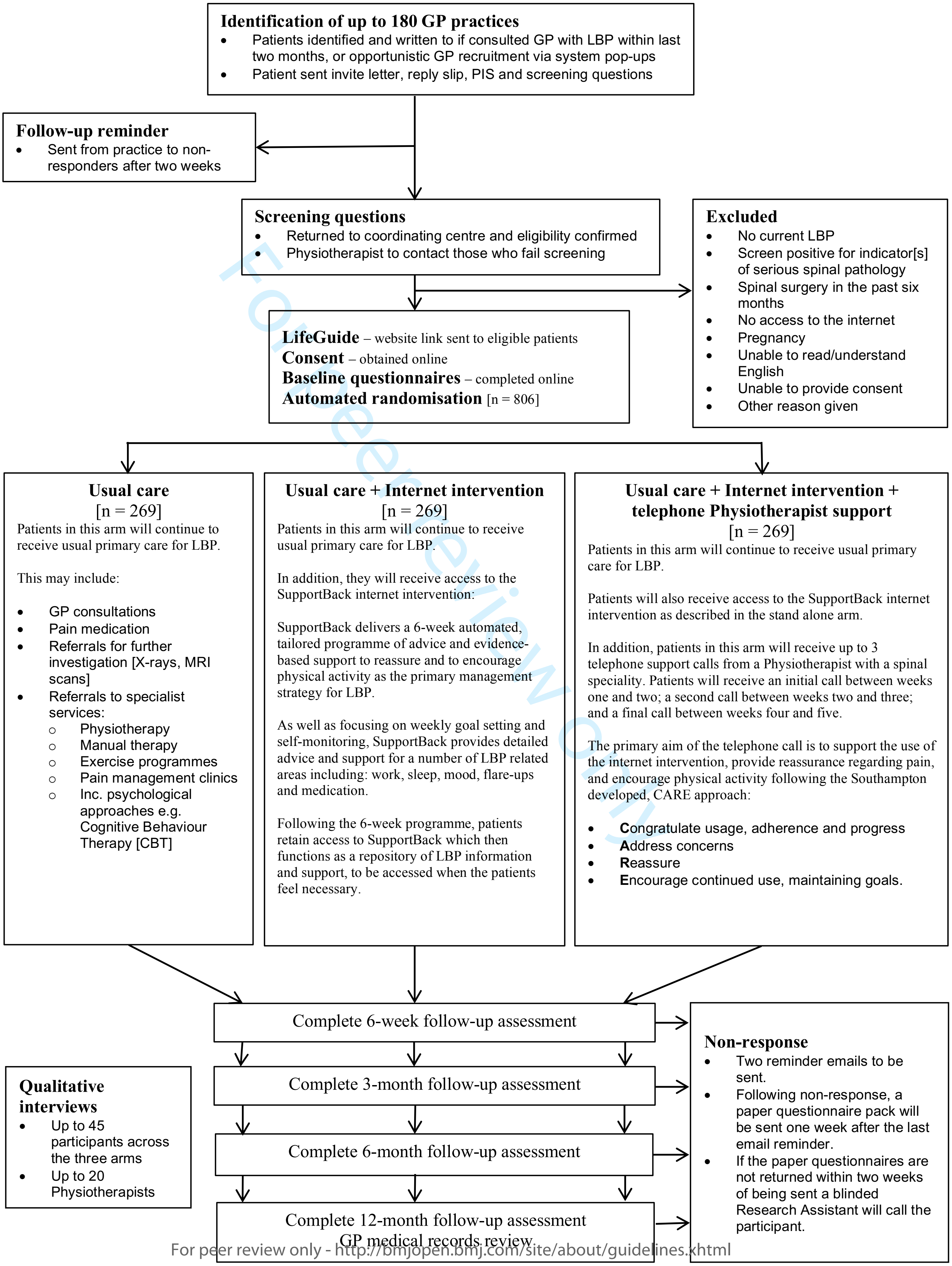

SPIRIT

STANDARD PROTOCOL ITEMS: RECOMMENDATIONS FOR INTERVENTIONAL TRIALS

SPIRIT 2013 Checklist: Recommended items to address in a clinical trial protocol and related documents*

Section/item	Item No	Description
**Administrative information**		
Title	1	Descriptive title identifying the study design, population, interventions, and, if applicable, trial acronym. **See page 1**
Trial registration	2a	Trial identifier and registry name. If not yet registered, name of intended registry. **See page 2**
	2b	All items from the World Health Organization Trial Registration Data Set. **See page 2, All can be found on ISRCTN register website.**
Protocol version	3	Date and version identifier. **N/A. This can be added if necessary.**
Funding	4	Sources and types of financial, material, and other support. **See page 20-21.**
Roles and responsibilities	5a	Names, affiliations, and roles of protocol contributors. **See page 1 for names and affiliations and page 20 for contributors.**
	5b	Name and contact information for the trial sponsor. **This can be added in necessary.**
	5c	Role of study sponsor and funders, if any, in study design; collection, management, analysis, and interpretation of data; writing of the report; and the decision to submit the report for publication, including whether they will have ultimate authority over any of these activities. **See page 20-21.**
	5d	Composition, roles, and responsibilities of the coordinating centre, steering committee, endpoint adjudication committee, data management team, and other individuals or groups overseeing the trial, if applicable (see Item 21a for data monitoring committee). **See page 18-19.**
**Introduction**		
Background and rationale	6a	Description of research question and justification for undertaking the trial, including summary of relevant studies (published and unpublished) examining benefits and harms for each intervention. **See page 4-5.**
	6b	Explanation for choice of comparators. **See page 4-5.**

For peer review only

Objectives	7	Specific objectives or hypotheses. **See page 4-5.**
Trial design	8	Description of trial design including type of trial (eg, parallel group, crossover, factorial, single group), allocation ratio, and framework (eg, superiority, equivalence, noninferiority, exploratory). **See page 5.**

Methods: Participants, interventions, and outcomes

Study setting	9	Description of study settings (eg, community clinic, academic hospital) and list of countries where data will be collected. Reference to where list of study sites can be obtained. **See page 5.**
Eligibility criteria	10	Inclusion and exclusion criteria for participants. If applicable, eligibility criteria for study centres and individuals who will perform the interventions (eg, surgeons, psychotherapists). **See page 5-6.**
Interventions	11a	Interventions for each group with sufficient detail to allow replication, including how and when they will be administered. **See page 8-10.**
	11b	Criteria for discontinuing or modifying allocated interventions for a given trial participant (eg, drug dose change in response to harms, participant request, or improving/worsening disease). **N/A**
	11c	Strategies to improve adherence to intervention protocols, and any procedures for monitoring adherence (eg, drug tablet return, laboratory tests). **See page 16-18.**
	11d	Relevant concomitant care and interventions that are permitted or prohibited during the trial. **See page 8-10.**
Outcomes	12	Primary, secondary, and other outcomes, including the specific measurement variable (eg, systolic blood pressure), analysis metric (eg, change from baseline, final value, time to event), method of aggregation (eg, median, proportion), and time point for each outcome. Explanation of the clinical relevance of chosen efficacy and harm outcomes is strongly recommended. **See page 10-12.**
Participant timeline	13	Time schedule of enrolment, interventions (including any run-ins and washouts), assessments, and visits for participants. A schematic diagram is highly recommended (see Figure). **See Figure 1.**
Sample size	14	Estimated number of participants needed to achieve study objectives and how it was determined, including clinical and statistical assumptions supporting any sample size calculations. **See page 13.**
Recruitment	15	Strategies for achieving adequate participant enrolment to reach target sample size. **See page 6-7.**

Methods: Assignment of interventions (for controlled trials)

Allocation:

For peer review only - http://bmjopen.bmj.com/site/about/guidelines.xhtml

Sequence generation	16a	Method of generating the allocation sequence (eg, computer-generated random numbers), and list of any factors for stratification. To reduce predictability of a random sequence, details of any planned restriction (eg, blocking) should be provided in a separate document that is unavailable to those who enrol participants or assign interventions. **See page 7-8.**
Allocation concealment mechanism	16b	Mechanism of implementing the allocation sequence (eg, central telephone; sequentially numbered, opaque, sealed envelopes), describing any steps to conceal the sequence until interventions are assigned. **See page 7-8.**
Implementation	16c	Who will generate the allocation sequence, who will enrol participants, and who will assign participants to interventions. **See page 7-8.**
Blinding (masking)	17a	Who will be blinded after assignment to interventions (eg, trial participants, care providers, outcome assessors, data analysts), and how. **See page 7-8.**
	17b	If blinded, circumstances under which unblinding is permissible, and procedure for revealing a participant's allocated intervention during the trial. **N/A.**
**Methods: Data collection, management, and analysis**		
Data collection methods	18a	Plans for assessment and collection of outcome, baseline, and other trial data, including any related processes to promote data quality (eg, duplicate measurements, training of assessors) and a description of study instruments (eg, questionnaires, laboratory tests) along with their reliability and validity, if known. Reference to where data collection forms can be found, if not in the protocol. **See page 13-14.**
	18b	Plans to promote participant retention and complete follow-up, including list of any outcome data to be collected for participants who discontinue or deviate from intervention protocols. **See page 13-14.**
Data management	19	Plans for data entry, coding, security, and storage, including any related processes to promote data quality (eg, double data entry; range checks for data values). Reference to where details of data management procedures can be found, if not in the protocol. **See page 13-14.**
Statistical methods	20a	Statistical methods for analysing primary and secondary outcomes. Reference to where other details of the statistical analysis plan can be found, if not in the protocol. **See page 14-15.**
	20b	Methods for any additional analyses (eg, subgroup and adjusted analyses). **See page 14-15.**
	20c	Definition of analysis population relating to protocol non-adherence (eg, as randomised analysis), and any statistical methods to handle missing data (eg, multiple imputation). **See page 14-15.**

Methods: Monitoring

Data monitoring	21a	Composition of data monitoring committee (DMC); summary of its role and reporting structure; statement of whether it is independent from the sponsor and competing interests; and reference to where further details about its charter can be found, if not in the protocol. Alternatively, an explanation of why a DMC is not needed. **See pages 18-19.**
	21b	Description of any interim analyses and stopping guidelines, including who will have access to these interim results and make the final decision to terminate the trial. **See pages 18-19.**
Harms	22	Plans for collecting, assessing, reporting, and managing solicited and spontaneously reported adverse events and other unintended effects of trial interventions or trial conduct. **See pages 18-19.**
Auditing	23	Frequency and procedures for auditing trial conduct, if any, and whether the process will be independent from investigators and the sponsor. **See pages 18-19.**

Ethics and dissemination

Research ethics approval	24	Plans for seeking research ethics committee/institutional review board (REC/IRB) approval. **See page 19.**
Protocol amendments	25	Plans for communicating important protocol modifications (eg, changes to eligibility criteria, outcomes, analyses) to relevant parties (eg, investigators, REC/IRBs, trial participants, trial registries, journals, regulators). **Can be added at Editors request?**
Consent or assent	26a	Who will obtain informed consent or assent from potential trial participants or authorised surrogates, and how (see Item 32). **See page 7.**
	26b	Additional consent provisions for collection and use of participant data and biological specimens in ancillary studies, if applicable. **N/A**
Confidentiality	27	How personal information about potential and enrolled participants will be collected, shared, and maintained in order to protect confidentiality before, during, and after the trial. **See page 18-19.**
Declaration of interests	28	Financial and other competing interests for principal investigators for the overall trial and each study site. **See page 21.**
Access to data	29	Statement of who will have access to the final trial dataset, and disclosure of contractual agreements that limit such access for investigators. **See page 21.**
Ancillary and post-trial care	30	Provisions, if any, for ancillary and post-trial care, and for compensation to those who suffer harm from trial participation. **N/A.**

For peer review only - http://bmjopen.bmj.com/site/about/guidelines.xhtml

Dissemination policy	31a	Plans for investigators and sponsor to communicate trial results to participants, healthcare professionals, the public, and other relevant groups (eg, via publication, reporting in results databases, or other data sharing arrangements), including any publication restrictions
	31b	Authorship eligibility guidelines and any intended use of professional writers. **N/A**
	31c	Plans, if any, for granting public access to the full protocol, participant-level dataset, and statistical code. **N/A**
**Appendices**		
Informed consent materials	32	Model consent form and other related documentation given to participants and authorised surrogates
Biological specimens	33	Plans for collection, laboratory evaluation, and storage of biological specimens for genetic or molecular analysis in the current trial and for future use in ancillary studies, if applicable

*It is strongly recommended that this checklist be read in conjunction with the SPIRIT 2013 Explanation & Elaboration for important clarification on the items. Amendments to the protocol should be tracked and dated. The SPIRIT checklist is copyrighted by the SPIRIT Group under the Creative Commons "Attribution-NonCommercial-NoDerivs 3.0 Unported" license.

For peer review only
