# Supplementary material for: Supporting self-management of low back pain with an internet intervention in primary care: a protocol for a randomised controlled trial of clinical and cost-effectiveness (SupportBack 2)
Source: BMJ Open. 2020 Aug 20;10(8):e040543. doi: 10.1136/bmjopen-2020-040543 (PMC7440707; doi:10.1136/bmjopen-2020-040543)
Supplement: Reviewer comments [file bmjopen-2020-040543.reviewer_comments.pdf]

PEER REVIEW HISTORY

BMJ Open publishes all reviews undertaken for accepted manuscripts. Reviewers are asked to complete a checklist review form (**http://bmjopen.bmj.com/site/about/resources/checklist.pdf**) and are provided with free text boxes to elaborate on their assessment. These free text comments are reproduced below.

ARTICLE DETAILS

**TITLE (PROVISIONAL)**	Supporting self-management of low back pain with an internet intervention in primary care: A protocol for a randomised controlled trial of clinical and cost-effectiveness (SupportBack 2)
**AUTHORS**	Geraghty, Adam; Roberts, Lisa; Hill, Jonathan; Foster, Nadine; Yardley, Lucy; Hay, Elaine; Stuart, Beth; Turner, David; Griffiths, Gareth; Webley, Frances; Durcan, Lorraine; Morgan, Alannah; hughes, Stephanie; Bathers, Sarah; Butler-Walley, Stephanie; Wathall, Simon; Mansell, Gemma; Leigh, Linda; Little, Paul

This article was not externally reviewed at BMJ Open. Protocols that have been independently assessed prior to submission to BMJ Open are usually fast-tracked to publication on the grounds that further substantial changes will not be possible. This independent assessment will usually be external, independent review for both a substantial grant award from a non-commercial or government funder and ethics approval.
